# The Impact of Exercise Training on the Brain and Cognition in Type 2 Diabetes, and its Physiological Mediators: A Systematic Review

**DOI:** 10.1186/s40798-025-00836-7

**Published:** 2025-04-24

**Authors:** Jitske Vandersmissen, Ilse Dewachter, Koen Cuypers, Dominique Hansen

**Affiliations:** 1 Faculty of Rehabilitation Sciences, REVAL - Rehabilitation Research Center, Hasselt University, Wetenschapspark 7, 3590 Diepenbeek, Belgium; 2 Biomedical Research Institute, BIOMED, Hasselt University, 3590 Diepenbeek, Belgium; 3 Movement Control and Neuroplasticity Research Group, Department of Movement Sciences, Group Biomedical Sciences, KU Leuven, 3000 Leuven, Belgium; 4 Heart Centre Hasselt, Jessa Hospital, 3500 Hasselt, Belgium

## Abstract

**Background:**

Type 2 diabetes (T2DM) affects brain structure and function, and is associated with an increased risk of dementia and mild cognitive impairment. It is known that exercise training has a beneficial effect on cognition and brain structure and function, at least in healthy people, but the impact of exercise training on these aspects remains to be fully elucidated in patients with T2DM.

**Objective:**

To determine the impact of exercise training on cognition and brain structure and function in T2DM, and identify the involved physiological mediators.

**Methods:**

This paper systematically reviews studies that evaluate the effect of exercise training on cognition in T2DM, and aims to indicate the most beneficial exercise modality for improving or preserving cognition in this patient group. In addition, the possible physiological mediators and targets involved in these improvements are narratively described in the second part of this review. Papers published up until the 14th of January 2025 were searched by means of the electronic databases PubMed, Embase, and Web of Science. Studies directly investigating the effect of any kind of exercise training on the brain or cognition in patients with T2DM, or animal models thereof, were included, with the exception of human studies assessing cognition only at one time point, and studies combining exercise training with other interventions (e.g. dietary changes, cognitive training, etc.). Study quality was assessed by means of the TESTEX tool for human studies, and the CAMARADES tool for animal studies.

**Results:**

For the systematic part of the review, 22 papers were found to be eligible. 18 out of 22 papers (81.8%) showed a significant positive effect of exercise training on cognition in T2DM, of which two studies only showed significant improvements in the minority of the cognitive tests. Four papers (18.2%) could not find a significant effect of exercise on cognition in T2DM. Resistance and endurance exercise were found to be equally effective for achieving cognitive improvement. Machine-based power training is seemingly more effective than resistance training with body weight and elastic bands to reach cognitive improvement. In addition, BDNF, lactate, leptin, adiponectin, GSK3β, GLP-1, the AMPK/SIRT1 pathway, and the PI3K/Akt pathway were identified as plausible mediators directly from studies investigating the effect of exercise training on brain structure and function in T2DM. Via these mediators, exercise training induces multiple beneficial brain changes, such as increased neuroplasticity, increased insulin sensitivity, and decreased inflammation.

**Conclusion:**

Overall, exercise training beneficially affects cognition and brain structure and function in T2DM, with resistance and endurance exercise having similar effects. However, there is a need for additional studies, and more methodological consistency between different studies in order to define an exercise program optimal for improving cognition in T2DM. Furthermore, we were able to define several mediators involved in the effect of exercise training on cognition in T2DM, but further research is necessary to unravel the entire process.

**Supplementary Information:**

The online version contains supplementary material available at 10.1186/s40798-025-00836-7.

## Key points

1. The current body of literature demonstrates a positive effect of exercise training on the brain in T2DM, but simultaneously emphasizes the need for additional studies on this topic.
2. BDNF, lactate, leptin, adiponectin, GSK3β, GLP-1, the AMPK/SIRT1 pathway, and the PI3K/Akt pathway have been identified as factors mediating the effect of exercise on the brain in T2DM.
3. There is a need for a standardised cognitive test battery to investigate the effect of exercise on cognition in T2DM.

**Supplementary Information:**

The online version contains supplementary material available at 10.1186/s40798-025-00836-7.

## Introduction

### Cognitive Decline in Aging

Cognitive decline is inherent to aging. As we age, synaptic plasticity reduces, neural mitochondrial function declines, epigenetic changes occur, etc., all contributing to decreased cognitive abilities [1–5]. Especially fluid intelligence, defined as the ability to solve problems, process new information, and learn new things, declines with aging. Fluid intelligence consists of several domains, including executive function, psychomotor ability, memory, and processing speed [6, 7].

Aging is associated with a decline in both grey and white matter volume, accompanied by ventricular enlargement and cortical thinning, as well as a decline in brain function [7, 8]. Amyloid β plaques are not only found in Alzheimer’s disease (AD) patients, but also in 20–30% of healthy adults, and are speculated to contribute to this neuronal loss [6, 9]. Also neuronal volume and the number of neuronal connections decreases due to a decline in the complexity of dendrite arborization, decreased neuritic spines, and reduced dendrite length [6, 10]. In addition, demyelination and decreased white matter integrity contribute to functional impairment of the brain [8].

### Cognitive Dysfunction in T2DM

T2DM patients are prone to more severe cognitive decline than what occurs during normal aging. A large fraction (45%) of T2DM patients experience cognitive dysfunction and have an increased risk of dementia [11–16]. Subjects with diabetes have a relative risk of 1.46 for AD, and a relative risk of 1.51 for any form of dementia [17]. Especially executive functioning, memory, attention, and information processing speed have been shown to be affected in T2DM [14, 18, 19]. This cognitive impairment is related to the affected brain structure and metabolism in T2DM. Decreased regional grey matter volume as well as altered intrinsic activity in the default mode network have been demonstrated in these patients [20–22]. Multiple pathophysiologies have been suggested as the cause of this, including cardiovascular complications, chronic low-grade inflammation, and hyperglycemia [14, 15, 18].

Several studies have demonstrated a link between T2DM and AD, also called type 3 diabetes. These studies focus on the fact that insulin signalling also plays an important role in the brain, and that T2DM and AD seem to share a number of pathophysiological processes such as amyloid β plaques, disturbed cerebral glucose metabolism, tau hyperphosphorylation, and inflammation [18, 23–26]. Insulin and insulin-like-growth-factor (IGF)-1 are important for neuronal survival and brain function. Numerous insulin receptors (IR) and IGF-1 receptors (IGF-1R) can be found in the brain, with a large amount being present in the hippocampus. One of the major downstream pathways of the IR is the PI3K/Akt pathway, which plays an important role in the regulation of brain function, and in the inactivation of GSK3β, which is an enzyme able to phosphorylate tau at pathological tau epitopes [23, 27, 28]. Reduced cerebral IR activation and insulin levels have been demonstrated in AD [25, 29], highlighting similar processes in AD and T2DM. Moreover, insulin-degrading enzyme is capable of clearing amyloid β peptides that aggregate into amyloid plaques, a key pathological hallmark of AD. In T2DM, competitive binding of high insulin concentrations and amyloid β peptides with this enzyme could be involved in decreased amyloid β clearance, thereby potentially contributing to increased accumulation of amyloid β peptides, and formation of amyloid β plaques [15, 30, 31].

### Positive Effect of Exercise on Cognitive Function

In healthy people, exercise has a positive effect on cognitive function [32–34]. This effect is mediated by multiple processes. Muscle contraction during exercise releases myokines, which stimulate the production of neurotrophic factors such as brain-derived neurotrophic factor (BDNF), promoting neurogenesis. Additionally, the exercise-induced release of anti-inflammatory factors contributes to balancing the brain’s redox status, counteracting many pathological processes [35–38]. Research shows that exercise reduces the age-related decrease in hippocampal volume [39–41], which, in combination with increased levels of neurotrophic factors, contributes to the maintenance of memory and neuroplasticity [36, 42, 43]. Moreover, there is a positive association between cardiorespiratory fitness levels and whole-brain and white matter volume in patients with early-stage AD [44]. In this way, exercise reduces the risk of dementia and neurodegenerative diseases [36, 45–47].

Lactate has been identified as an important contributor to the positive effect of exercise on cognitive function. During anaerobic exercise, lactate is released from contracting muscles, eventually leading to increased lactate levels in the brain after crossing the blood–brain-barrier (BBB). This results in an increased expression of brain plasticity genes such as BDNF, Arc, and c-fos, contributing to neurogenesis [48–50]. In addition, lactate also binds to hydroxycarboxylic acid receptor 1 (HCAR1), which leads to an increase in vascular endothelial growth factor (VEGF), stimulating angiogenesis and thereby contributing to increased cognitive function [50–52]. Also the myokine irisin has been shown to positively influence cognition [53]. Synaptic plasticity and memory in amyloid pathology mimicking AD mouse models can be rescued by boosting brain levels of FNDC5/irisin, and peripheral overexpression of FNDC5/irisin rescues memory impairment [54]. Cathepsin b is another myokine which has been associated with improved cognition. It is known to play a role in both neurogenesis and angiogenesis [55, 56]. Cathepsin b levels increase in mouse and human plasma in response to exercise, which is positively correlated with memory, while cathepsin b-knockout mice do not show any cognitive improvements following running exercise [57]. This suggests that cathepsin b is mandatory for the positive effect of exercise on cognition.

### The Association of Physical Activity with Cognitive Function in T2DM

Because of the above-mentioned positive effects of exercise on cognitive function, and to get more insight into whether this translates to T2DM, some studies have explored the association between physical activity (PA) and cognition in T2DM. The effect of exercise training on the brain in T2DM could be different than in other populations due to the reduced insulin sensitivity in this patient group. Exercise increases insulin sensitivity, and could thus have a more pronounced effect in insulin resistant patients [58, 59]. Moreover, the existing cognitive decline in T2DM [11, 12] could affect the extent of the effect of exercise on cognition in patients with T2DM. In addition, cardiovascular complications such as atherosclerosis can increase the risk of vascular dementia in T2DM [60, 61], and are responsive to exercise training [62, 63].

For example, one study found significantly higher cognitive scores in an active group of T2DM patients compared to a sedentary group of T2DM patients [64]. They also found a significant negative correlation (r = − 0.2, *p* = 0.03) between cognition and BMI in the sedentary group, and a significant positive correlation (r = 0.55, *p* = 0.01) between cognition and minutes of weekly exercise in the active group. Another study found that active elderly T2DM patients had a significantly slower rate of cognitive decline compared to non-active/sedentary elderly T2DM patients, more specifically in the global cognition (*p* = 0.005), executive functioning (*p* = 0.014), and attention/working memory (*p* = 0.01) domains [65]. These findings were confirmed by multiple other studies [66–68].

In a study investigating the influence of the BDNF Val66Met polymorphism on the association between cognition and PA in diabetic patients, it was found that carriers of the Met-allele showed significantly higher scores of words recall (*p* < 0.001), mental status (*p* = 0.004), and total cognition, (*p* = 0.04), and had a significantly higher education level. Overall, PA was associated with better total cognition, words recall, and mental status, no matter the intensity. This association was strongest in the Met/Met carrier group, and stronger for females than for males within this group. They also found that light to moderate PA showed greater association with cognitive domains than moderate to vigorous PA. These findings thus suggest that female Met/Met diabetic carriers cognitively benefit the most from PA, and that light to moderate PA has the most influence on the brain [68].

Another study examined the individual and joint influence of diabetes status, apolipoprotein E (APOE) ε4, and PA on the risk of dementia and cognitive impairment without dementia (CIND) in cognitively normal older adults. The risk of dementia and CIND was higher in diabetic patients and APOE ε4 carriers who reported low levels of moderate to vigorous PA. The risk was even found to be nearly tenfold higher for physically inactive diabetic APOE ε4 carriers. Higher levels of PA were associated with lower dementia/CIND risk [67].

Overall, these findings suggest that higher levels of PA are associated with better cognitive function and less cognitive decline in T2DM. However, based on these studies alone, one cannot assume causality. In addition, there is a possibility of reverse causality, where reduced cognitive function contributes to lower levels of PA. Therefore, several studies have investigated the acute effect of exercise on cognitive function in T2DM.

### The Acute Effect of Exercise on Cognitive Function in T2DM

The acute effect of exercise on cognition in T2DM has mainly been investigated in the domain of executive function.

One study found that both endurance and resistance training acutely improved inhibitory control and response time in T2DM patients [69]. In addition, moderate-intensity integrated concurrent exercise (ICE), consisting of both endurance and resistance training, acutely improved all three aspects of executive function (inhibition, conversion and refresh function) in cognitively normal hospitalised T2DM patients, while resistance exercise improved inhibition, and endurance exercise only caused significant improvements in the refresh function. The ICE-induced improvements in executive function were accompanied by a simultaneous increase in cerebral blood flow in the dorsolateral prefrontal cortex (DLPFC), the frontal pursuit area (FPA), and the orbitofrontal cortex (OFC), while resistance exercise showed a corresponding activation of DLPFC and FPA, and endurance exercise increased cerebral perfusion in the FPA, OFC and Broca region [70]. These results suggest that an exercise-induced increase in cerebral blood flow in certain areas can elicit specific cognitive improvements. This knowledge can be used to customise exercise programs in T2DM patients in order to obtain the desired effect.

These studies already give some insight into the effect of exercise on cognition in T2DM. However, with the aim to incorporate exercise in T2DM treatment for cognitive improvement, it is necessary to know the effect of *long-term exercise training* on the brain, which can be different from the acute effect. Therefore, this review will focus on pre-post intervention studies. This review only describes RCTs and clinical trials investigating the effect of exercise training on cognition and brain structure in T2DM and animal models thereof, with special emphasis on the mediators and targets involved in this. So far, this is the first review of this kind. The knowledge provided by this review could more reliably provide insights in the mechanisms of cognitive decline and cognitive improvement in T2DM, and could be used to design more specific exercise programs to target the brain in T2DM patients.

## Methods

This review consists of two parts, the first of which is a systematic review of the papers investigating the effect of exercise training on cognition in T2DM. The second part narratively describes studies that show beneficial brain changes or brain changes in combination with cognitive improvement in T2DM as a result of exercise training, and aims to identify the involved mediators by pooling studies discussing similar pathways or targets. The addition of this second part distinguishes this review from previous similar reviews [71–73], and deepens the understanding of how exercise improves cognition, and identifies pathways that could be pharmacologically targeted to mimic the effect of exercise on cognition in individuals unable to exercise.

### Eligibility

The PICO (population, intervention, comparison, outcome) method was used to determine whether articles were eligible for inclusion in this review. In order to be included in the systematic part of this review, the articles had to be written in English, and had to investigate the effect of any kind of exercise training on cognition in T2DM patients or T2DM animal models. Studies investigating the effect of a single exercise bout or physical activity during daily living were not included. Exercise training in T2DM was compared to usual care, or a different kind (intensity/modality/…) of exercise training in T2DM. Studies without a T2DM control group were not included. Articles discussing only type 1 diabetes were excluded, as well as articles only investigating depression, anxiety, or other mental disorders in T2DM. Also studies investigating the effect of exercise training in combination with another intervention (e.g. dietary changes, cognitive training, etc.) were excluded. Studies in which cognitive function was only assessed at one time point in human subjects were excluded since these make it impossible to accurately assess the effect of exercise training on cognition (pre- and post-intervention assessments mandatory). The narrative part of this review includes studies from the systematic section that also examined certain cerebral pathways, mediators, or targets. Additionally, to further explain the findings, we also reference other studies that explore the effects of exercise on these cerebral pathways, mediators, or targets in T2DM, even if they do not directly assess cognition.

### Study Selection

**Fig. 1:**
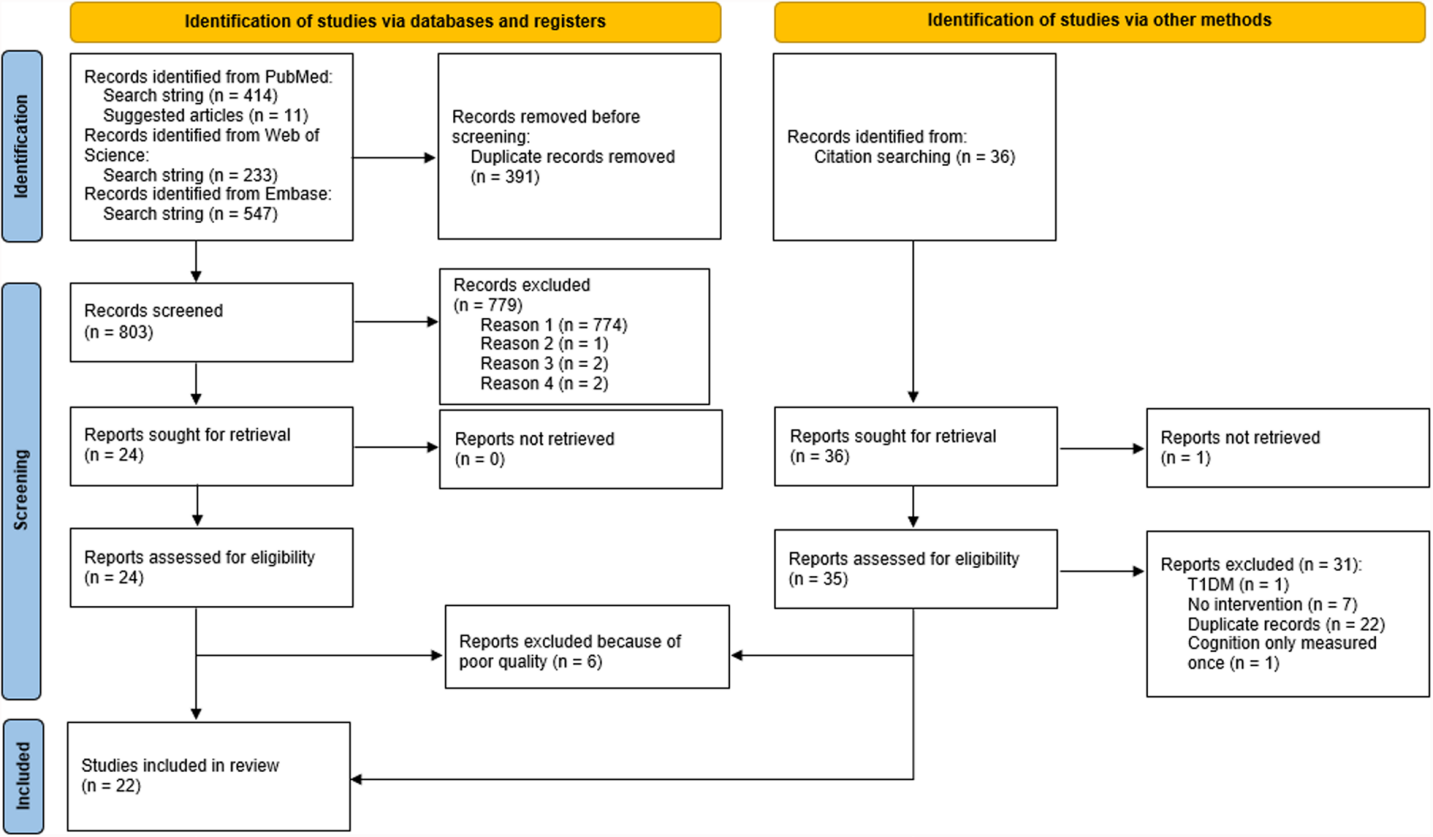
Prisma flow chart of study selection procedure. Reason 1: Does not discuss the effect of exercise training on cognition in T2DM; Reason 2: Article not available in English; Reason 3: Article only discusses the protocol. Study is not conducted yet. Reason 4: No T2DM control group. T1DM = type 1 diabetes. From: Page MJ, McKenzie JE, Bossuyt PM, Boutron I, Hoffmann TC, Mulrow CD, et al. The PRISMA 2020 statement: an updated guideline for reporting systematic reviews. BMJ 2021;372:n71. https://doi.org/10.1136/bmj.n71

The papers described in the systematic part of this review were found by entering the following search string into PubMed, Embase and Web of Science: (exercise OR "physical activity" OR "aerobic exercise" OR "aerobic training" OR "resistance exercise" OR "resistance training" OR "combined exercise" OR "combined training" OR “endurance exercise” OR “endurance training”) AND (T2DM OR diabetes OR "type 2 diabetes") AND (memory OR "executive function*" OR "processing speed" OR attention OR brain OR cognition OR "cognitive function"). The filters “randomised controlled trial” and “clinical trial” were applied. Relevant related articles suggested by PubMed when accessing articles found by entering the aforementioned search string were also considered. Reference lists of reviews in line with the present one [71–79] were examined as well. The current review discusses articles published up until the 14th of January 2025. Duplicate articles (identical PMID) and studies not meeting the TESTEX or CAMARADES criteria were removed (Fig. 1). The following information was extracted from the studies: author(s), year of publication, age of the subjects, type of subjects (population), number of subjects in the intervention group, type of control group, number of subjects in the control group, type, duration, frequency, and intensity of the exercise intervention, duration of each exercise session, outcome measures (cognitive tests), and main cognitive results. Effect sizes were extracted, or calculated if not mentioned. For the narrative part of the review, only studies directly investigating the effect of exercise on the brain in T2DM were considered to identify plausible mediators and pathways.

### Quality Control

Quality control of the included human studies was conducted by means of the Tool for the assEssment of Study qualiTy and reporting in EXercise (TESTEX), which is designed specifically for exercise training studies [80]. With this tool, a maximal score of 15 can be reached, with 5 points assigned to study quality, and 10 points to reporting. A score of ≥ 7/15 was required for inclusion (Table 1). Quality of the animal studies was assessed by means of the Collaborative Approach to Meta-Analysis and Review of Animal Data from Experimental Studies (CAMARADES) checklist [81]. Animal studies with a score of < 6/10 were excluded (Table 2). If nothing was mentioned about the criterion, it was indicated as not fulfilled.

**Table 1:** TESTEX characteristics of included human studies

	1	2	3	4	5	6	6a	6b	6c	7	8	8a	8b	9	10	11	12	Total
Leischik et al. [82]	√	√	√	√	X	X	/	/	/	X	√	√	√	√	X	X	X	7/15
Ploydang et al. [83]	√	√	√	√	X	√	√	√	X	√	√	√	√	√	X	X	√	11/15
Wang et al. [84]	√	√	√	√	√	X	/	/	/	X	√	√	√	√	√	X	X	9/15
Zhao et al. [85]	√	√	√	X	√	√	X	√	√	√	√	√	√	√	X	√	√	12/15
Yamamoto et al. [86]	√	√	X	√	X	√	√	√	√	X	√	√	√	√	X	X	X	9/15
Teixeira et al. [87]	√	√	X	√	X	X	/	/	/	√	√	√	√	√	√	√	√	10/15
Silveira-Rodrigues et al. [88]	√	X	X	√	X	√	X	X	√	√	√	√	√	√	√	√	√	10/15
Espeland et al. [89]	√	√	X	√	√	√	X	√	√	X	√	√	√	√	X	X	X	9/15
Silveira-Rodrigues et al. [90]	√	X	X	√	X	X	/	/	/	X	√	√	√	√	X	√	√	7/15
Ghodrati et al. [91]	√	√	X	√	X	√	√	X	X	X	√	√	√	√	X	√	√	9/15
Martinez-Velilla et al. [92]	√	√	√	√	√	√	√	√	X	√	√	√	√	√	√	X	X	12/15
Callisaya et al. [93]	√	√	√	√	√	√	√	√	√	√	√	√	√	√	X	X	X	12/15
Ghahfarrokhi et al. [94]	√	√	√	√	√	√	√	√	√	√	√	√	√	√	X	X	√	13/15
Chen et al. [95]	√	√	X	√	√	√	√	√	√	√	√	√	√	√	X	X	X	11/15
Cai et al. [96]	√	X	X	√	X	√	√	√	X	X	√	√	√	√	X	X	X	7/15
Liu et al. [97]	√	√	√	√	√	X	/	/	/	X	√	√	X	√	X	X	√	8/15

√ = fulfilled, X = not fulfilled, 1 = Eligibility criteria specified, 2 = Randomisation specified, 3 = Allocation concealed, 4 = Groups similar at baseline, 5 = Blinding of assessor, 6 = Outcome measures assessed in 85% of patients, 6a = adherence > 85%, 6b = adverse events reported, 6c = exercise attendance reported, 7 = Intention-to-treat analysis, 8 = Between-group statistical comparisons reported, 8a = between-group statistical comparisons reported for primary outcome measure of interest, 8b = between-group statistical comparisons reported for at least one secondary outcome measure, 9 = Point measures and measures of variability for all reported outcome measures, 10 = Activity monitoring in control groups, 11 = Relative exercise intensity remained constant, 12 = Exercise volume and energy expenditure

**Table 2:** CAMARADES characteristics of included animal studies

	1	2	3	4	5	6	7	8	9	10	Total
Parsa et al. [98]	√	√	√	X	√	√	√	X	√	√	8/10
Shekarchian et al. [99]	√	√	√	X	X	√	√	X	X	√	6/10
Jesmin et al. [100]	√	√	√	X	X	√	√	X	X	√	6/10
Lang et al. [101]	√	√	√	√	√	√	√	X	√	√	9/10
Shima et al. [102]	√	√	√	X	X	√	√	X	√	√	7/10
Shima et al. [103]	√	√	X	X	X	√	√	X	√	√	6/10

√ = fulfilled, X = not fulfilled, 1 = Published in peer-reviewed journal, 2 = Control of temperature, 3 = Randomisation, 4 = Allocation concealed, 5 = Blinding of assessors, 6 = No anaesthetics with marked intrinsic properties, 7 = Use of animals with diabetes, 8 = Sample size calculation, 9 = Compliance with regulatory requirements, 10 = Statement regarding conflict of interest

## Results

### Study Selection

See Fig.1.

### Quality Control

See Table 1.

### Systematic Review on the Effect of Exercise Training on Cognition in T2DM

#### Endurance Exercise Training

##### Human Studies

Endurance exercise as simple as walking can already significantly improve cognition in T2DM patients. In a study by Leischik et al. (2021), T2DM patients were randomised into a walking group, pedometer group or control group. The walking group had to walk for 40 min, 3 times a week. The pedometer group had to reach 10,000 steps per day. Both the walking group and the pedometer group, the latter of which managed to reach an average of 8700 steps per day, showed significant improvements in attention and non-verbal memory. Verbal memory also significantly improved in the walking group [82]. In contrast, a pilot RCT on the effect of 3 months of walking at moderate intensity on cognitive function in T2DM failed to find significant improvements in executive function, episodic memory, working memory, and processing speed [97]. Besides normal walking, also aquatic Nordic walking performed for 60 min 3 times a week for 12 weeks, has proven to cause significantly increased Montreal Cognitive Assessment (MoCA) scores in T2DM patients. However, no significant improvements were seen in the MMSE, trail making test part B, or Stroop colour and word test [83].

Wang et al. (2023) tested the effect of outdoor aerobic dancing on hippocampal volume and cognition in 82 T2DM patients. The patients were divided into a control group and a training group. After one year of endurance training, they found significantly increased MMSE and MoCA scores in the training group compared to the control group, suggesting that endurance exercise can improve cognitive function in T2DM [84].

##### Animal Studies

Also animal studies show a predominantly positive effect of endurance exercise training on cognition. Shekarchian et al. (2023) found that streptozotocin- and high fat diet-induced diabetic C57BL/6 J mice that received 4 weeks of swimming training scored significantly better on tests for working, spatial, and recognition memory compared to non-exercising diabetic mice [99]. Another study showed that swimming training for 12 weeks in streptozotocin-nicotinamide-induced type 2 diabetic rats improved exploratory behaviour, locomotor activity, passive avoidance memory, and non-spatial cognitive memory compared to their sedentary counterparts. However, this improvement was found to be insignificant [98].

The Morris water maze test has oftentimes been used to demonstrate the effect of endurance exercise on cognition in animal models of T2DM. In this test, the animal is placed in a circular swimming pool where a platform is hidden somewhere under the water surface. After a few practice rounds, the time it takes the animal to find the platform, and the length of the path that is followed, are measured. These measures give an indication of spatial learning and memory [104]. For example, one study looked into the effect of 4 months of both light and moderate intensity treadmill running on memory in pre-symptomatic Otsuka-Long-Evans-Tokushima fatty (OLETF) rats. Before the exercise intervention, the OLETF rats spent considerably less time in the quadrant area where the platform was located during the learning phase, compared to control Long-Evans Tokushima (LETO) rats. After the intervention, both exercise intensities showed to have improved memory function in the OLETF rats, since both escape latency and swim length were shortened [100]. Lang et al. (2020) also used the Morris water maze test to test the effect of 8 weeks of moderate-intensity treadmill exercise on memory in T2DM mice. The exercising T2DM mice showed a significantly reduced escape latency on day 4–5 compared to the non-exercising T2DM mice, and their number of platform crossings significantly increased over time [101]. Another similar study was performed where the effect of 4 weeks of moderate-intensity treadmill running in OLETF rats was investigated. They found that both swim path length and escape latency of the exercising OLETF rats were reduced at trials on days 2–4, and that their time spent in the platform area significantly improved after the exercise intervention [102]. Another study by Shima et al. (2023) assessed the effect of 4 weeks of light-intensity running on a forced exercise wheel bed in obese-hyperglycemic (ob/ob) mice. However, here they did not find an effect of exercise on the swim distance, escape latency, or speed. They did find that the times of crossing the target platform during the probe test in the exercised ob/ob mice did not differ significantly from that in the control C57BL/6 mice, and that it was greater than in the sedentary ob/ob mice, but not significantly [103].

Based on these studies, endurance exercise training conducted for 40–60 min, 3 times per week seems to enable cognitive improvements in T2DM patients. Especially memory shows to be sensitive to endurance exercise-induced improvement. However, two out of four human studies discussed here only found significant improvements in MoCA and/or MMSE scores. Since the MMSE and MoCA were designed as screening tools to diagnose mild cognitive impairment (MCI) [105, 106], they cannot be considered very reliable for detecting changes in cognitive function.

#### Resistance Exercise Training

##### Human Studies

The beneficial effect of resistance exercise on cognition in T2DM patients was shown by Zhao et al. (2022). They examined the effect of 12 months of power training on executive function, attention/speed, memory, and global cognition in older adults with T2DM, and aimed to determine whether there is an association between cognitive improvements and improvements in muscle strength, body composition, and/or endurance. 103 patients were divided into a power training group or a sham low-intensity group that performed the same exercises as the power training group, but without added weight. Cognition of both groups improved over time, with increased scores in the Trails A, Trails B, word list recall, and word list memory. In addition, improved memory was associated with both increased skeletal muscle mass and reduced body fat mass, and improvement in Trails B minus A in the power training group was associated with increases in knee extension strength [85]. Yamamoto et al. (2021) failed to find similar results. In their study, 60 T2DM patients aged 72.9 ± 2.4 years were divided into a control group, a resistance training group, and a resistance training group with leucine (an amino acid promoting muscle synthesis) supplementation. The training groups performed daily bodyweight resistance exercises and exercises with elastic bands every day at home, for a total period of 48 weeks. Cognitive function was assessed by means of the MMSE at baseline and after the intervention period. The MMSE score in the control group was significantly decreased to 27.5 ± 2.6 after 48 weeks, while the score in the training groups had not significantly changed. This caused the MMSE score at 48 weeks to be significantly higher in the training groups than in the control group [86].

Both studies suggest a positive effect of resistance exercise training on cognition in T2DM, with machine-based resistance exercise training being the most effective. However, a sufficient number of studies to draw a definite conclusion is lacking. Moreover, the study of Yamamoto et al. only assessed cognitive function by means of the MMSE, limiting the information on cognitive function, and thus the reliability of the results.

#### Endurance Versus Resistance Exercise Training

##### Human Studies

To determine the exercise modality most effective in improving cognition in T2DM, some studies compared the effect of endurance and resistance exercise training. Teixeira et al. (2029) investigated the effect of either endurance or resistance exercise on cognitive function in T2DM patients or patients with arterial hypertension. The patients were randomised into a resistance exercise group or an endurance exercise group. Both groups exercised at moderate intensity for 12 weeks. Cognitive function was assessed before and after the intervention by means of the mental test and training system (MTTS), consisting of the cognitrone (attention and concentration), the determination test (reaction time), and the visual pursuit test (selective attention). The group with both T2DM and hypertension, but not the group with only hypertension, showed a significantly improved performance in the cognitrone, but no significantly improved reaction time. No differences were observed between the endurance and resistance exercise group. These results show that both endurance and resistance exercise are capable of increasing attention and concentration in T2DM patients with arterial hypertension [87].

This study shows that endurance and resistance exercise training have a comparable positive effect on cognitive function in T2DM, however, additional evidence confirming these results is needed.

#### Combined Endurance and Resistance Exercise Training

##### Human Studies

Several studies have explored the effect of combined exercise training, consisting of both resistance and endurance exercise, sometimes in combination with flexibility or balance exercises, on cognition in T2DM patients. One study found significantly improved inhibitory control, working memory, cognitive flexibility, and attention after 8 weeks of combined exercise training in T2DM patients, compared to a control group [88]. The same research team also examined the effect of 8 weeks of combined exercise on plasma BDNF levels, executive function and long-term memory in T2DM patients. They observed significantly improved executive function following the combined exercise, while BDNF levels were not found to be significantly changed [90]. Espeland et al. (2027) conducted an exploratory analysis of data from the Lifestyle Interventions and Independence for Elders (LIFE) trial, which was a randomised controlled clinical trial of exercise intervention consisting of walking, resistance training, and flexibility exercises in sedentary non-demented T2DM patients and healthy subjects. Cognitive function was tested at baseline and 2 years after randomisation. They found that cognitive function, more specifically global cognitive function and delayed memory, significantly improved in the diabetic participants of the intervention group only, suggesting a beneficial effect of combined training on cognition in T2DM [89]. Additionally, one study determined the effect of 12 weeks of combined exercise training, consisting of endurance, resistance, and balance exercises, on cognition in women with T2DM. Cognition was tested by means of the MoCA, the digit symbol substitution test, and the forward digit span test. After the 12-week intervention, the exercise group scored significantly higher on the MoCA compared to the control group, and, with an increase of 3.1, improved significantly compared to baseline. However, neither of the groups showed improvement in the other tests [91].

In a study by Martinez-Velilla et al. (2021), 103 acutely hospitalised elderly T2DM patients were randomised to an exercise group or a control group. Cognitive function was assessed by means of the MMSE at baseline and at discharge. The exercise intervention consisted of a combination of resistance, balance, and walking exercises. The median length of stay, and thus of the intervention, was 8 days. There was no difference in MMSE score between both groups at baseline. However, after the intervention, the exercise group scored on average 1.6 points higher than the control group (23.7 vs. 22.1), which was found to be significant [92]. In addition, a pilot study looked into the effect of 6 months of a progressive endurance- and resistance-training program on the brain and cognition in T2DM. 50 T2DM patients were randomised into an intervention group and a control group. The intervention group performed 6 months of endurance and progressive resistance training. The control group received upper and lower limb stretching of light intensity and a gentle movement program, which were performed in the same volume, frequency and setting as in the intervention group. The intervention group showed improved hippocampal and total brain volumes, improved white matter integrity, and less decline in white matter volume. They also showed a better global cognitive score, and better performance on the Digit Symbol Coding Test, Rey Complex Copy test, Stroop C-D, Trail Making Test A and B, Hopkins verbal learning test (intermediate and recognition scores), and Controlled Oral Word Association Test compared to the control group [93]. Another pilot study examined the effect of 6 weeks of high-intensity low-volume (HIFT) vs. low-intensity high-volume (LIFT) functional training on cognition in cognitively impaired elderly T2DM patients. The HIFT group exercised three times a week at 100–120% of the lactate threshold, while the LIFT group exercised five times a week at 70–75% of the lactate threshold. The MMSE was used to diagnose cognitive impairment (MMSE ≤ 23). Processing speed, learning, memory, and attention were assessed by means of the Symbol Digit Modalities Test (SDMT), California Verbal Learning Test Second Edition (CVLT-II), Brief Visuospatial Memory Test-Revised (BVMT-R), and Stroop tests respectively. After the intervention, MMSE, Stroop, SDMT, CVLT-II and BVMT-R scores had improved significantly in the HIFT group, while only MMSE and Stroop scores had improved significantly in the LIFT group. However, the only cognitive score change that was significantly different from the control group that did not receive an exercise intervention, was the change in Stroop scores in the HIFT group [94].

All included studies suggest a positive effect of combined exercise training on cognitive function in T2DM.

#### Other Types of Exercise

##### Human Studies

Besides the well-known exercise forms such as running and cycling, some studies have explored the effect of unconventional exercise training on cognition in T2DM.

For example, one study compared the effect of 36 weeks of Tai Chi Chuan, a mind–body exercise, and 36 weeks of fitness walking on global cognitive function in T2DM patients. Both interventions significantly increased MoCA scores, with a significantly larger effect of Tai Chi Chuan compared to fitness walking. At 24 weeks and 36 weeks, the Weschler memory quotient (MQ), digit symbol substitution test, and trail making test part B scores also improved significantly more in the Tai Chi Chuan group than in the control group. At 36 weeks, the MQ scores even improved significantly more in the Tai Chi Chuan group compared to the fitness walking group [95].

The effect of low-intensity Qigong exercise on cognitive function in older T2DM patients was also investigated [96]. Qigong is an ancient Chinese exercise for mind–body integration and is often used in the prevention and treatment of chronic metabolic diseases [107]. The participants in the exercise group practiced Kinect-based Kaimai-style Qigong for 12 weeks. Based on MMSE scores at baseline and after the intervention, the Qigong group showed significant improvements in cognitive function compared to the control group [96].

These findings suggest that unconventional exercise, often involving the mind to a larger extent than conventional exercise, can contribute to improved cognition in T2DM.

### Reviewing Targets and Mediators of the Effect of Exercise Training on the Brain in T2DM

To understand why certain exercise trainings cause cognitive improvement in T2DM, and others do not, there is a need for studies investigating the underlying biomedical mechanisms of the effect of exercise on the brain and cognition in T2DM. The following section discusses such studies, and aims to identify the different targets and mediators involved.

#### AD-Related Pathological Markers: Amyloid β Peptides and Hyperphosphorylated Tau

Increasing evidence and literature points to converging pathways and pathogenetic processes in T2DM and AD. Here we focus on studies showing effects on amyloid β and tau in T2DM due to exercise training. We refer to extensive reviews regarding T2DM and AD for further reading [24, 108, 109].

##### Leptin

Several studies have indicated that exercise is negatively associated with cerebral amyloid β and hyperphosphorylated tau in T2DM, and contributes to memory maintenance [110, 111]. The aspartyl protease β-site AβPP-cleaving enzyme 1 (BACE1) is responsible for catalysing the rate-limiting step of amyloid β production. It has been shown that the adipocytokine leptin reduces BACE1 activity and expression, thereby limiting the production of amyloid β [112, 113]. This is supported by Rezaei et al. (2023), who suggested leptin as a possible mediator of exercise-induced prevention of memory impairment in T2DM after finding elevated serum and hippocampal levels of this hormone together with decreased hippocampal levels of BACE1, amyloid β and hyperphosphorylated tau after 8 weeks of HIIT training in T2DM rats [114].

##### GSK3β

The maintenance of cognitive function in exercising T2DM rats treated with dexamethasone, demonstrated by De Sousa et al. (2020), was according to the authors presumably mediated by the observed lesser inhibition of the activation of hippocampal IRS-1 and higher concentration of GSK3β phosphorylated on serine-9 (Ser-9) [115]. GSK3β is namely inactivated by Ser-9 phosphorylation [116], which prevents GSK3β activation, and subsequently tau phosphorylation and associated neurotoxic effects [117, 118]. Accordingly, Rezaei et al. (2023) also found decreased hippocampal GSK3β dephosphorylation together with decreased hippocampal levels of amyloid β and phosphorylated tau in HIIT-trained T2DM rats [114]. GSK3β plays an important role in neurodegeneration in T2DM. Both glucolipotoxicity and insulin resistance contribute to GSK3β overactivation, leading to β-catenin phosphorylation and subsequent proteasomal degradation. This results in the inhibition of the expression of reactive oxygen species (ROS) scavenging enzymes, which leads to more oxidative stress and consequently the disruption of mitochondrial structure, function and axonal trafficking. Moreover, GSK3β overactivation also stimulates tau hyperphosphorylation, leading to microtubule destabilisation and thus disruption of axonal mitochondrial trafficking, as well as to a decrease in mitochondrial complex I and thus impaired energy production [118]. By inhibiting the activation of GSK3β through Ser-9 phosphorylation, exercise could thus contribute to the prevention of cognitive decline in T2DM.

##### Adiponectin

In the study of Rezaei et al. (2023), the T2DM rats showed decreased serum and hippocampal levels of insulin and adiponectin at baseline, as well as decreased levels of insulin receptors, adiponectin receptors and AMPK in the hippocampus, and increased hippocampal GSK3β and hyperphosphorylated tau. HIIT training counteracted these findings. Both serum insulin and adiponectin were significantly increased by HIIT, as well as the levels of hippocampal insulin and adiponectin receptors. HIIT training also increased hippocampal AMPK phosphorylation, and decreased hippocampal tau phosphorylation and GSK3β dephosphorylation. The authors suggest that the increased levels of adiponectin can contribute to the preservation of hippocampal volume and function through the stimulation of synaptic plasticity, as it has been shown by Pousti et al. (2018) and Weisz et al. (2017) respectively that adiponectin modulates synaptic plasticity in the hippocampal dentate gyrus and regulates hippocampal synaptic transmission [114, 119, 120]. Another study shows memory and learning impairments together with reduced AMPK phosphorylation and increased GSK3β activation in adiponectin-knockout mice [121], suggesting that the neuroprotective effects of adiponectin are mediated via AMPK phosphorylation and GSK3β inactivation, which is in line with the study of Rezaei et al. (2023). The exercise-induced decreased GSK3β Ser-9 dephosphorylation means that there is a higher ratio of inactive, phosphorylated GSK3β, preventing tau phosphorylation. In addition, the adiponectin-mediated AMPK phosphorylation could also be neuroprotective by inhibiting the inflammatory response of microglia to amyloid β [122].

#### Brain Volume

Several studies have demonstrated that exercise increases hippocampal volume in T2DM patients. One study showed that a higher step count, measured over 7 days, was associated with a larger hippocampal volume in T2DM patients [66]. In addition, one year of endurance training in T2DM patients with normal cognition significantly increased hippocampal volume, and prevented a decline in MMSE and MoCA scores. Hippocampal volume in the training group was significantly increased compared to baseline, as well as compared to the control group [84]. Another study showed a higher hippocampal CA1 and CA3 neuronal density in diabetic Sprague–Dawley (SD) rats after 6 weeks of exercise on a running wheel, probably contributing to the observed improved cognitive function [123]. Similarly, a pilot study in which T2DM patients performed 6 months of a progressive endurance- and resistance-training program showed improved hippocampal and total brain volumes, improved white matter integrity, and less decline in white matter volume in the intervention group compared to the control group [93].

#### Neuronal Survival

##### BDNF

BDNF is one of the most important neurotrophic factors. By binding the TrkB receptor, it regulates and promotes cell survival, neuroplasticity, and neurogenesis in the central nervous system [124, 125]. As already mentioned previously, BDNF plays an important role in the effect of exercise on cognitive function. A study investigating the effect of 4 weeks of swimming training on working, spatial, and recognition memory in diabetic C57BL/6 J mice, shows that higher PA is associated with increased hippocampal and prefrontal BDNF levels, together with an improvement in all memory aspects [99]. Similarly, Jesmin et al. (2022) showed significantly increased hippocampal BDNF levels together with improved memory function in OLETF rats after 4 months of endurance exercise [100]. This shows that also under diabetic conditions, BDNF is an important mediator of exercise effects on cognition.

Another study looked into the effect of 12 weeks of swimming training on BDNF/TrkB signalling and apoptosis in the cerebral cortex of male diabetic C57BL/6JNarl mice. The exercised diabetic mice showed less neural apoptosis compared to the control diabetic mice, accompanied by an increased activity of the BDNF/TrkB signaling pathway, and a decreased activity of the Fas/FasL-mediated and mitochondria-initiated apoptotic pathways. These findings indicate that exercise promotes neuronal survival in diabetic mice through the BDNF pathway [126].

#### Neuroplasticity and Neurogenesis

One study determined the effect of 7 weeks of treadmill exercise on neuroblast differentiation in the subgranular zone of the dentate gyrus (SZDG) in Zucker diabetic fatty (ZDF) rats and Zucker lean control (ZLC) rats. At 23 weeks old, the rats started to exercise for 7 weeks at 12-16 m/min. Compared to the non-exercising groups, the neuroblasts of both the exercising diabetic and exercising control rats showed a significant increase in tertiary dendrites. However, the number of neuroblasts only increased in the control rats. This shows that endurance exercise in diabetic rats can stimulate neuroplasticity, but not necessarily neuroproliferation [127]. A similar study tested the effect of 5 weeks of treadmill exercise on cell differentiation and proliferation in the SZDG of the same ZDF rat model. Proliferation was detected by means of the proliferation marker Ki67, and progenitor differentiation into neurons was detected by means of the differentiation marker doublecortin (DCX). In this study, the rats started exercising at 6 weeks old, and ran at 22 m/min. Compared to the sedentary ZDF rats, the exercised rats showed a significant increase in Ki67 positive cells and DCX-immunoreactive structures, indicating both increased proliferation and increased differentiation [128]. This confirms that exercise can stimulate neuronal differentiation in the diabetic SDZG, as shown by Hwang et al. (2010) [127]. The fact that, this time, exercise was able to stimulate neuronal proliferation as well, might indicate that exercise has to be performed at a certain intensity rather than a certain volume to achieve this effect.

##### BDNF

Increased hippocampal BDNF levels in combination with increased dendritic spine density on the secondary and tertiary dendrites of dentate granule neurons were found after voluntary wheel running in db/db mice [129]. This suggests that BDNF contributes to exercise-induced neuroplasticity in diabetic mouse models.

##### PI3K/Akt and AMPK/SIRT1

It was demonstrated that endurance exercise can upregulate synaptic plasticity-associated proteins in the hippocampus of T2DM rats, presumably via the activation of PI3K/Akt/mTOR and AMPK/SIRT1 signalling pathways and inhibition of the NFκB/NLRP3/IL-1β signalling pathway [130]. Under non-diabetic conditions, the PI3K/Akt/mTOR pathway is activated by insulin, resulting in the formation of dendritic spines and excitatory synapses in hippocampal neurons [131]. In T2DM, insulin resistance prevents this activation, contributing to reduced synaptic plasticity. Exercise thus could, to a certain extent, take over this role of insulin. The aforementioned BDNF also activates the PI3K/Akt pathway, constituting another mediator of the exercise-induced increase in synaptic plasticity-associated proteins [124]. Activation of the AMPK/SIRT1 pathway, on the other hand, contributes to synaptic plasticity by increasing insulin sensitivity and glucose uptake, regulating BDNF expression, promoting neuronal survival, etc. [132–136]. During exercise, AMPK activates SIRT1, after which SIRT1 deacetylates PGC-1α, resulting in its activation. PGC-1α is important for mitochondrial remodelling and biogenesis, contributing to synaptic plasticity [137]. Its activation leads to the upregulation of FNDC5, which in turn crosses the BBB and upregulates hippocampal BDNF expression [124].

Another study also found an increase in the synaptic plasticity proteins synaptophysin (SYN) and N-methyl-D-aspartate receptor (NMDAR) in the prefrontal cortex of diabetic SD rats after 4 weeks of treadmill training. They demonstrated that endurance exercise increased the level of phosphorylated PI3K, suggesting an increased activity of the PI3K/Akt pathway, and increased insulin sensitivity. In addition, they found a slight increase in the phosphorylation and a slight decrease in the acetylation of FOXO1, which is a transcription factor that promotes NF-kB activity and consequently the expression of proinflammatory factors. FOXO1 phosphorylation enables its cytoplasmic retention and thus prevents the transcription of its target inflammatory genes [138]. Since FOXO is a target of Akt, the increased PI3K/Akt activity might be the cause of the increased FOXO1 phosphorylation [139]. Nuclear NF-kB acetylation was found to be decreased as well in the rats that performed endurance exercise, reducing its DNA binding and transcriptional activity [138, 140]. These findings were confirmed by a study showing that 8 weeks of moderate intensity treadmill exercise in diabetic C57BL/ 6 mice improves cognitive dysfunction and increases the release of BDNF through the activation of the SIRT1/PGC-1α/FNDC5/BDNF pathway [101].

#### Cerebral Blood Flow

Zhao et al. (2023) investigated the effect of a 2-month moderate-intensity exercise program on brain oxygenation in sedentary older T2DM patients with cognitive impairment. They also looked into the effect of the exercise program on the hemodynamic responses to the Mini-Cog test, which assesses short-term memory and visuo-constructive abilities. The exercise program consisted of a combination of endurance and resistance exercise. They found that the exercise program improved the very low-frequency oscillations (< 0.05 Hz) during the Mini-Cog test, as assessed by near-infrared spectroscopy. Since very-low frequency oscillations are crucial for small-vessel function, this indicates that combined exercise has the power to improve cerebral vascular function in T2DM. In addition, the exercise program significantly reduced the oxyhemoglobin activation in the right superior frontal region during the Mini-Cog test, thereby reducing the overactivation usually observed in T2DM [141].

#### Glycometabolism

##### Lactate

Since dysregulated peripheral glycometabolism is a hallmark of T2DM, Shima et al. (2017) hypothesised that hippocampal glycometabolic dysfunction might contribute to memory impairment in T2DM [102]. As it has already been demonstrated in healthy animals that moderate exercise enhances memory function and hippocampal glycogen levels [142, 143], they wanted to determine if this was also the case in a T2DM animal model. At baseline, OLETF rats had higher hippocampal levels of glycogen and lower MCT2 expression compared to LETO control rats. They also showed impaired memory. After 4 weeks of treadmill running, the OLETF rats showed even higher hippocampal glycogen levels, in addition to normalised hippocampal MCT2 expression together with improved memory function. Since MCT2 is an important lactate transporter, this suggests that lactate plays a key role in the effect of exercise on glycometabolism and memory in T2DM [102]. In line with this, it was demonstrated that 4 months of treadmill running prevented the progression of cognitive decline in presymptomatic OLETF rats through improved hippocampal MCT2 expression, again confirming the role of lactate in the positive effect of exercise on cognition [100]. Another study by Shima et al. (2023) showed the same effect in an advanced stage T2DM mouse model. Ob/ob mice were subjected to 4 weeks of light-intensity exercise. The exercise program resulted in improved hippocampal MCT2 levels, accompanied by improved hippocampal memory retention [103]. As Soya et al. (2019) mention in their review discussing these findings, the upregulated MCT2 levels enable an increased uptake of L-lactate into neurons, where it can serve as an energy substrate, and act as a signalling molecule to induce the expression of neuroplasticity genes, eventually contributing to the increased memory function observed in these studies [144].

#### Inflammation

As inflammation is a very broad process, involving many different players, we would like to emphasize again that the pathways and mediators discussed in this review are only the ones that clearly have been shown to be involved in the effect of exercise on the brain in T2DM. For more information about the involvement of inflammation in T2DM, and the effect of exercise on it, we refer to more exhaustive reviews [78, 145].

##### AMPK/SIRT1

The AMPK/SIRT1 pathway is not only important for rescuing cognitive function by increasing BDNF levels, but also by counteracting inflammation. Treadmill exercise in diabetic C57BL/ 6 mice for 8 weeks reduced activation of hippocampal proinflammatory microglia M1, as well as the hippocampal levels of proinflammatory factors IL-1β, IL-6, TNF-α, and increased the expression levels of anti-inflammatory factors IL-10, TGF-β1. This co-occurred with an activation of the SIRT1/NF-κB pathway, which is known to be responsible for counteracting inflammation, suggesting the importance of this pathway in exercise-mediated anti-inflammatory actions in T2DM [101]. A different study also reported a reduction in hippocampal IL-1β and TNF-α, accompanied by cognitive improvement, after T2DM SD rats performed 6 weeks of endurance exercise on a running wheel. However, although we can assume that the AMPK/SIRT1 pathway is involved here as well, the authors did not elaborate on the pathways responsible for the observed reduction in pro-inflammatory factors [123].

#### Insulin Resistance

T2DM-associated insulin resistance is not only present in the periphery, but also in the brain, where it contributes to the cognitive deficits observed in T2DM patients via multiple mechanisms [132, 146–148]. For example, insulin resistance and the consequent hyperinsulinemia have been associated with increased tau hyperphosphorylation, increased deposition together with reduced breakdown of amyloid β, dysfunctional IGF-1 receptors (IGFRs), etc. This all contributes to neurodegeneration and cognitive impairment [146]. Studies have shown that the insulin-sensitising effect of exercise is also manifested in the brain, which gives exercise the power to prevent or decrease these cognitive deficits [58, 149]. One possible way in which this effect of exercise could be mediated, is by the aforementioned decrease in TNF-α. TNF-α activates JNK, which is a kinase that causes insulin resistance via serine phosphorylation of IRS-1 [78, 150]. IRS-1 is important for the signal transduction of insulin, ultimately leading to glycogen synthesis and glucose transporter 4 (GLUT-4) translocation. Serine phosphorylation of IRS-1 inhibits this insulin signal transduction and thus results in insulin resistance [151–153]. It could thus be hypothesised that by decreasing TNF-α, exercise could increase insulin sensitivity in the brain.

##### GLP-1

Park et al. (2019) investigated the effect of resistance exercise training on GLP-1R levels in the hypothalamus of OLETF rats. The exercise training comprised 12 weeks of ladder climbing. As a result of the resistance training, the rats showed increased levels of hypothalamic GLP-1R mRNA, protein kinase A (PKA), protein kinase B (PKB), glucose transporter 2 (GLUT-2), and decreased hypothalamic levels of protein kinase C-iota (PKC-ι) [154]. All these findings point in the direction of improved glycaemic control. PKB, also known as Akt, plays an important role in the PI3K/Akt pathway downstream of the IR, as mentioned before in this review [23, 27, 130]. PKA, on the other hand, is essential for the regulation of metabolism and triglyceride storage [155, 156], while PKC-ι is known to cause metabolic abnormalities in T2DM [157, 158]. GLP-1R, the main focus of this study, is a main receptor involved in T2DM. It is responsible for lowering blood glucose levels by, among other things, stimulating insulin secretion and suppressing glucagon secretion [159]. In addition, GLP-1R has been suggested to play a neuroprotective role in diabetes-related neurodegeneration by increasing insulin sensitivity [160]. Moreover, a different study has shown that recombinant human GLP-1 can reduce oxidative stress by activating PKA, which was found to be elevated in the study of Park et al. (2019), and by inhibiting PKC, which was found to be decreased, ultimately reversing diabetic nephropathy [161]. The increase in GLP-1 mRNA found by Park et al. (2019) thus indicates decreased oxidative stress and increased insulin sensitivity in the T2DM rats after resistance exercise training.

#### Mitochondrial Function

Mitochondrial dysfunction is an important contributor to T2DM pathology [162, 163]. Multiple studies have already demonstrated mitochondrial dysfunction in the T2DM brain, where it contributes to neurodegeneration and cognitive dysfunction [164, 165]. It is also known that exercise positively influences mitochondrial function [166–168]. Studies show that exercise can restore mitochondrial function in the muscle of T2DM patients [169–172]. However, little is known about the effect of exercise on mitochondrial function in the brain in T2DM.

##### Insulin Sensitivity

One study investigated the effect of endurance exercise training in combination with metformin on mitochondrial function in male C57BL/6 J mice with brain insulin resistance induced by a high-fat diet. They observed that the mitochondria in brain regions rich in insulin receptors produced less ATP and showed reduced activity of oxidative enzymes. The mice also showed elevated ROS production and reduced activity of antioxidant enzymes, accompanied by higher rates of mitochondrial fission, and accumulation of damaged mitochondrial proteins. Endurance exercise training together with metformin improved insulin sensitivity. This resulted in reduced ROS emission, less hippocampal mitochondrial fission, less mitochondrial protein oxidation, and increased ATP production in astrocytes and primary cortical neurons. The reduction in ROS emission and increased ATP production were counteracted by intranasal administration of the insulin receptor antagonist S961, proving that these mitochondrial ameliorations were mediated by increased insulin sensitivity [173]. Since metformin was also administered in this study, the observed effects are not fully attributable to exercise, however, it is clear that metformin and exercise have a synergistic effect, and that exercise can certainly be an added value in the treatment of T2DM.

**Fig. 2:**
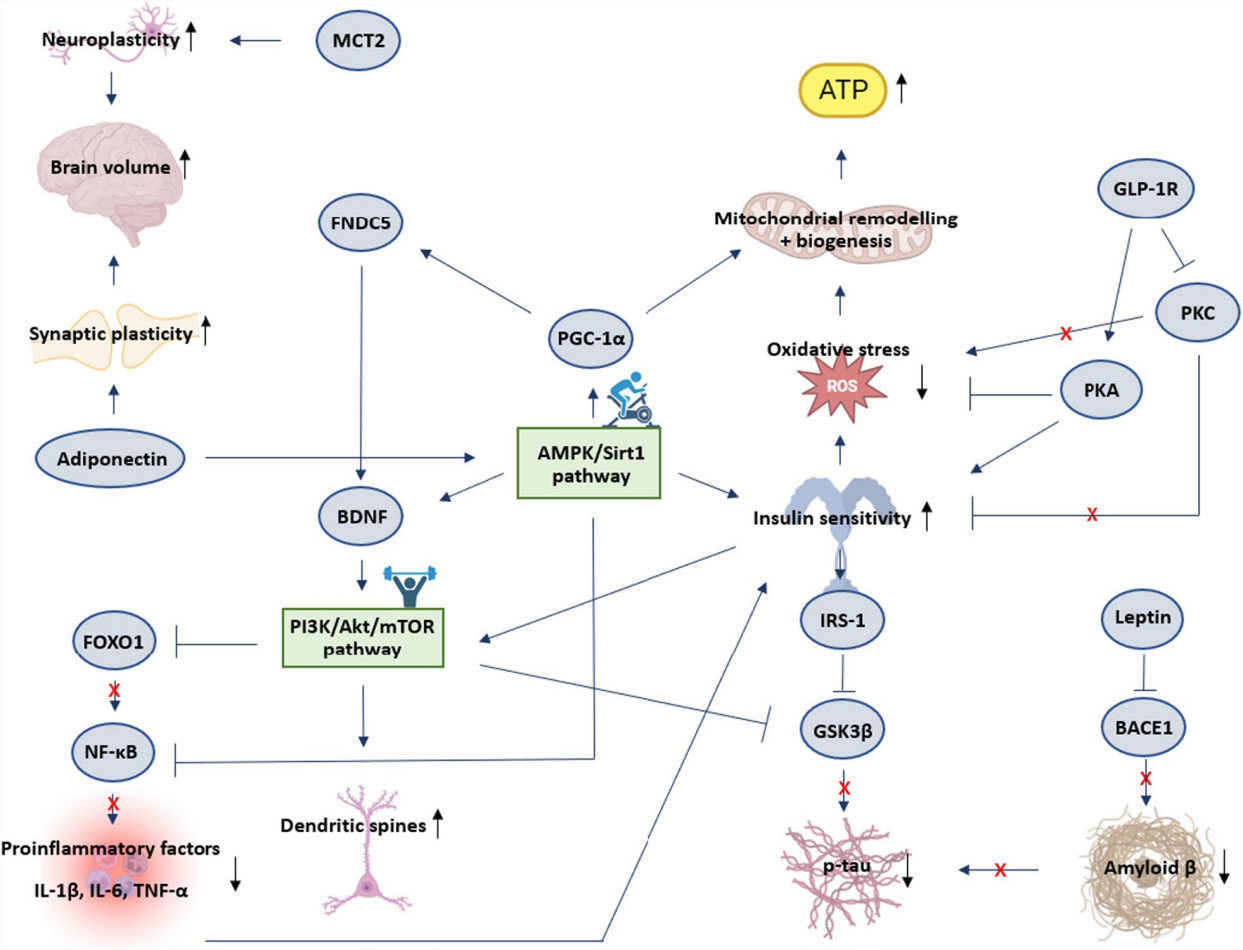
Schematic representation of mediators and targets involved in the effect of exercise training on the brain in T2DM. Sharp blue arrows indicate stimulation. Blunt blue arrows indicate inhibition. Black arrows indicate increase or decrease. A red cross indicates a mediator’s inability to exert an effect due to previous inhibition of said mediator. Aβ = amyloid β; AMPK = AMP-activated Protein Kinase; ATP = Adenosine Triphosphate; BDNF = Brain-Derived Neurotrophic Factor; FNDC5 = Fibronectin type III Domain-Containing protein 5; FOXO1 = Forkhead box protein O1; GLP-1R = Glucagon-Like Peptide 1 Receptor; GSK3β = Glycogen Synthase Kinase-3 beta; IRS-1 = Insulin Receptor Substrate 1; IL-1β = Interleukin 1beta; IL-6 = Interleukin 6; MCT2 = Monocarboxylate Transporter 2; mTOR = mammalian Target Of Rapamycin; Nf-κB = Nuclear Factor kappa-light-chain-enhancer of activated B-cells; PGC-1α = Peroxisome proliferator-activated receptor Gamma Coactivator 1-alpha; PI3K = Phosphoinositide 3-Kinase; PKA = Protein Kinase A; PKC = Protein Kinase C; p-tau = phosphorylated tau; ROS = Reactive Oxygen Species; Sirt1 = Sirtuin 1; TNF-α = Tumor Necrosis Factor alpha

The figure below (Fig. 2) gives an overview of the interplay between all mediators and pathways previously mentioned.

## Discussion

In the systematic part of this review, 22 studies investigating the effect of exercise training on cognition in T2DM were included. Although mixed results were found, most studies (18 out of 22) demonstrated a significant positive effect of exercise training on cognition, which is consistent with findings of previous reviews on this topic [72, 73].

7 out of 10 studies investigating the effect of endurance exercise training were able to show cognitive improvement, indicating the effectiveness of this training modality. Leischik et al. showed that endurance exercise as simple as walking is already successful in improving cognition in T2DM [82]. However, this was not confirmed by Liu et al., who could not find any significant cognitive improvements after a 3-month walking intervention [97]. A small sample size and a short follow-up period were mentioned as possible reasons for the lack of a significant effect. However, sample size, duration of the exercise intervention, and frequency and length of the individual exercise sessions were comparable to the study of Leischik et al., where significant improvements were seen. This might suggest that the walking intensity in the study of Leischik et al. was more optimal, although this information was not disclosed.

Out of the two studies investigating the effect of resistance exercise on cognition in T2DM, one study failed to show cognitive improvement. This lack of effect could be due to the limited cognitive tests, or due to the absence of machine-based power training in this exercise intervention. Additional studies are required to clarify this.

The study of Teixeira et al. comparing endurance exercise training to resistance exercise training found similar results for both exercise forms. This suggests that exercise interventions in T2DM patients aimed at improving cognition do not have to be limited to one type of exercise, and can be adapted to the patient’s capacities. However, since only one study directly comparing these exercise modalities was identified, this conclusion should be interpreted with caution.

In addition, all included studies investigating the effect of combined (endurance + resistance (+ flexibility/balance)) exercise training were able to show significant cognitive improvements. Being in line with the exercise recommendations of the American College of Sports Medicine for older adults [174], this finding favours combined exercise to reach cognitive improvement in T2DM.

Finally, tai chi chuan and Kaimai style Qigong showed to improve cognition in T2DM. The beneficial effect of these exercise forms might be attributed to the fact that they are motorically more complex, and require a more extensive involvement of the mind.

Based on this review, one can thus conclude that both endurance and resistance exercise training, as well as exercise training involving the mind, are effective at improving cognition in T2DM. Combined exercise training is presumably most desirable, although studies comparing combined exercise training to endurance or resistance exercise training alone are lacking. It also seems that regarding resistance exercise training, machine-based power training is more effective in improving cognition than body weight exercises and exercises with elastic bands, but additional evidence is required.

The beneficial effect of exercise on cognitive function in T2DM was confirmed by previous reviews on this topic [73, 79]. A meta-analysis by Cai et al. found a significant positive effect of exercise on global cognitive function in older adults with T2DM, without a significant influence of intervention modality or duration [73]. In contrast, several reviews could not conclude a significant positive effect of exercise on cognition in T2DM [71, 74]. Four out of the six studies included in the systematic review of Zhao et al. found significant benefits of exercise for some aspects of cognition in adults with type 2 diabetes, insulin resistance or impaired glucose tolerance, but only 26% of the cognitive outcomes were significant across all studies [71]. Also Cooke et al. found no significant effect of exercise on executive function or memory after conducting a meta-analysis of 6 studies on the effect of exercise on cognition in T2DM. [74]. These contradictory results emphasize the need for additional qualitative studies on this topic.

The mechanisms underlying cognitive improvement following exercise in T2DM have been explored in the second, narrative part of this review. Both the AMPK/Sirt1 pathway and PI3K/Akt/mTOR pathway have been identified as key players. The studies included in this review show that exercise-mediated activation of these pathways leads to beneficial brain changes in T2DM, such as increased neuroplasticity, brain volume, synaptic plasticity, dendritic spines, insulin sensitivity, mitochondrial remodelling and biogenesis, and ATP production, as well as decreased inflammation, oxidative stress, and amyloid β and p-tau production. BDNF, lactate, leptin, adiponectin, GSK3β and GLP-1 were identified as the most important factors mediating these changes. Although other factors such as IGF-1 have been suggested to play a role in the positive effect of exercise on cognition [175], no studies in T2DM have directly demonstrated this. Much more research is needed to identify the full spectrum of involved mediators and pathways, investigate interactions between different cell types, etc. In addition, it needs to be considered that the majority of the studies described in this review were conducted in rodents, and are yet to be confirmed in humans.

This review has various limitations that could interfere with the reliable interpretation of its findings. First of all, the fact that the studies included in this review use a variety of cognitive tests, makes it difficult to compare the different studies. Moreover, several studies merely use the MMSE or MoCA to test cognition, which are diagnostic tools designed to screen for MCI, and not to detect (subtle) changes in cognition [105, 106]. There is a need for a standardised cognitive test battery in T2DM to increase the comparability of future studies and draw more reliable conclusions. In addition, the age, medication use, and ethnicity of the patient population varied across studies. Since cognition is heavily influenced by age [176], and medication use [177, 178] and genetics [68] strongly affect the response to exercise, comparability of the included studies may have been limited by these inconsistencies. Regarding the narrative part of this review, an important limitation is that only studies directly demonstrating an involvement of certain mediators in the effect of exercise training on the brain in T2DM have been considered. This means the present review is a non-exhaustive presentation of the involved mediators and pathways. Finally, publication bias might have skewed the results of this review towards a positive effect of exercise on cognition in T2DM.

In order to define an exercise program that is specifically tailored to cognitive improvement in T2DM, future studies should directly compare exercise of different intensities and volumes, as well as different exercise modalities. In addition, as already mentioned, a standardised cognitive test battery should be used that is most sensitive to picking up exercise-induced cognitive changes in T2DM. Furthermore, medication use, disease stage, age, and genetics should be taken into account.

## Conclusion

Overall, it can be concluded that exercise has a positive influence on cognition and brain structure in T2DM. There are few studies that fail to find a positive correlation between exercise training and cognition in T2DM, which can often be attributed to a small sample size or a limited cognitive test battery. From this review, we can assume that resistance and endurance exercise training have similar effects on cognition in T2DM, with resistance exercise training seemingly requiring machine-based exercises. We can also conclude that unconventional exercise training involving the mind, such as tai chi, is capable of inducing cognitive improvements as well. However, since the cognitive tests and exercise training programs used in studies investigating the effect of exercise on cognition in T2DM is so diverse, it is difficult to compare these studies and draw a definite conclusion on the best exercise program for cognitive improvement in T2DM. Future studies should focus on using a standardised cognitive test battery, and comparing similar exercise training interventions where only one parameter (intensity/duration/frequency/…) differs.

## Supplementary Information

Additional file 1.

## Data Availability

The papers and associated datasets used for the current manuscript are available from PubMed, using the following search string: (exercise OR "physical activity" OR "aerobic exercise" OR "aerobic training" OR "resistance exercise" OR "resistance training" OR "combined exercise" OR "combined training" OR “endurance exercise” OR “endurance training”) AND (T2DM OR diabetes OR "type 2 diabetes") AND (memory OR "executive function*" OR "processing speed" OR attention OR brain OR cognition OR "cognitive function"). The following information was extracted from the studies: author(s), year of publication, age of the subjects, type of subjects (population), number of subjects in the intervention group, type of control group, number of subjects in the control group, type, duration, frequency, and intensity of the exercise intervention, duration of each exercise session, outcome measures (cognitive tests), and main cognitive results with their effect sizes. Exercise intervention characteristics, outcome measures and cognitive results can be found in Tables 3 and 4. Population characteristics are presented in supplementary Table 1 and 2 in the appendix.

**Table 3:** Intervention characteristics and outcomes of included human studies

	Author	Type of exercise training	Duration of exercise intervention	Duration of each training session	Frequency of exercise training	Intensity of exercise training	Outcomes (cognitive tests)	Main cognitive results	Effect size
Endurance exercise training	Leischik et al. [82]	Walking	12 weeks	40 min	3x/week	?	Cognitive attention/performance (FAIR-Test 2), verbal memory, non-verbal memory	Verbal memory, nonverbal memory, and cognitive performance significantly improved in the walking group and E-health group compared to the control group	Cognitive performance: Walking vs. control: d = 0.73 E-health vs. control: d = 1.15 Quality value: Walking vs. control: d = 0.34 E-health vs. control: d = 0.41 Continuity value: Walking vs. control: d = 0.63 E-health vs. control: d = 1.05 Verbal memory: Walking vs. control: d = 0.73 E-health vs. control: d = 1.70 Non-verbal memory: Walking vs. control: d = 6.57 E-health vs. control: d = 6.32
	Ploydang et al. [83]	Aquatic Nordic walking	12 weeks	60 min	3x/week	First 6 weeks: 40–50% HRR Last 6 weeks: 50–60% HRR	Visuospatial/executive function, naming, memory, attention, language, abstraction, delayed recall, and orientation (MoCA), orientation to time, orientation to place, registration, attention and calculation, word recall, language, and visual construction (MMSE), executive function (TMT part B), inhibition (Stroop colour and word test)	Significantly increased MoCA scores in Nordic walking group. No significant improvements in MMSE, Stroop test, or TMT	MoCA: d = 0.46 MMSE: d = 0.05 Stroop: d = 0.12 TMT B: d = 0.03
	Wang et al. [84]	Outdoor aerobic dancing	1 year	60 min	3x/week	?	Cognitive screening (MMSE) + cognitive impairment (MoCA)	Aerobic training group had significantly increased MMSE and MoCA scores compared to the control group	MMSE: d = 0.58 MoCA: d = 0.55
	Liu et al. [97]	Walking	3 months	50 min	3x/week	moderate intensity: 40–59% HRR	Executive function (dimensional change card sort test) Episodic memory (picture sequence memory test) Working memory (List sorting working memory test) Processing speed (Pattern Comparison Processing Speed Test)	Non-significant increases in executive function, episodic memory, working memory and processing speed scores	Executive function: η^2^ = 0.027 Episodic memory: η^2^ = 0.013 Working memory: η^2^ = 0.008 Processing speed: η^2^ = 0.045
Resistance exercise training	Zhao et al. [85]	Whole-body, machine based power training (3 sets of 8 reps of lateral pulldown, chest press, upper back, leg press, knee extension, and knee flexion, hip extension and hip abduction)	12 months	?	3 days/week	High intensity (15–18 on Borg scale, 80% 1RM)	Memory (word list memory, word list recall, and word list recognition subtests of the Consortium to Establish a Registry for Alzheimer’s Disease), attention/speed (TMT Part A (Trails A)), executive function: (TMT Part B (Trails B) + Trail Making Test B minus A (Trails B minus A)), global cognitive function (MMSE)	Cognition of both groups improved over time, with increased scores in the Trails A, Trails B, word list recall, and word list memory	MMSE: d = − 0.52 TMT A: d = 0.10 TMT B: d = 0.16 TMT B – TMT A: d = 0.31 Word list memory: d = − 0.04 Word list recall: d = 0.32 Word list recognition: d = 0.00
	Yamamoto et al. [86]	Body weight resistance training + exercises with elastic bands: tube fly, front raise, hammer curl, leg extension, calf raise, and squat Performed 20 times each	48 weeks	?	Daily	?	Cognitive function (MMSE)	No significant changes in MMSE score	MMSE: d = 0.59
Endurance vs resistance exercise training	Teixeira et al. [87]	Endurance exercise: elliptical, bike, treadmill, or upper-body cycle ergometer Resistance exercise	12 weeks	40 min	3x/week	Endurance: 60% HRmax resistance: 11–12 Borg scale	MTTS: attention and concentration(cognitrone), reaction time (determination test), selective attention (visual pursuit test)	The group with both T2DM and hypertension showed a significantly increased number of reactions, but no significantly improved reaction time. No differences were observed between the endurance and resistance exercise group	/
Combined endurance and resistance exercise training	Silveira-Rodrigues et al. [88]	6 resistance exercises (reverse grip lat pulldown, leg press, bench press machine, calf raises machine, dumbbell shoulder press, and abdominal crunches) + 15 min of treadmill walking, with increasing weight and repetition number over 8 weeks	8 weeks	?	3x/week	week 1–2: 20 min at 100% v6MWT (aerobic) 2 × 13–15 MRs, i = 60 s (strength) week 3–4 20 min at 105% v6MWT (aerobic) 3 × 13–15 MRs, i = 60 s (strength) week 5–6 25 min at 105% v6MWT (aerobic) 3 × 10–12 MRs, i = 60 s (strength) week 7–8 25 min at 105–110% v6MWT (aerobic) 3 × 8–10 MRs, i = 60 s (strength)	Cognitive screening (MoCA), visuospatial memory (Taylor's complex figure test), processing speed (TMT A), cognitive flexibility (TMT B), verbal fluency (semantic and alternate word fluency), processing speed (digit symbol substitution), working memory (digit span test), attention/concentration/inhibitory control (Stroop colour test)	Significantly improved inhibitory control, working memory, cognitive flexibility, and attention in the exercise group compared to the control group	Memory: d = − 0.07 Verbal fluency: d = − 0.01 Processing speed: d = − 0.10 Attention/concentration: d = 0.64 Cognitive flexibility: d = 0.67 Inhibitory control: d = 0.89 Working memory: d = 0.88
	Espeland et al. [89]	Walking, resistance training, and flexibility exercises	2 years	50 min	3–4x/week	Moderate intensity	Global cognitive function (3MSE), psychomotor speed, attention, and working memory (the Wechsler Adult Intelligence Scale-III DSC test), delayed recall (HVLT-D), processing speed and executive function (computer tests: 1-back and 2-back tasks, the Eriksen Flanker task, and a task-switching paradigm)	Global cognitive function and delayed memory, significantly improved only in T2DM patients who received training	3MSE: d = 3.24 DSC: d = 1.50 HVLT-D: d = 3.24 Executive function: d = 0.69
	Silveira-Rodrigues et al. [90]	6 resistance exercises (reverse grip lat pulldown, leg press, bench press machine, calf raises machine, dumbbell shoulder press, and abdominal crunches) + treadmill walking, with increasing weight and repetition number over 8 weeks	8 weeks	40–60 min	3x/week	week 1–2: 20 min at 100% v6MWT (aerobic) 2 × 13–15 MRs, i = 60 s (strength) week 3–4 20 min at 105% v6MWT (aerobic) 3 × 13–15 MRs, i = 60 s (strength) week 5–6 25 min at 105% v6MWT (aerobic) 3 × 10–12 MRs, i = 60 s (strength) week 7–8 25 min at 105–110% v6MWT (aerobic) 3 × 8–10 MRs, i = 60 s (strength)	Executive function: cognitive flexibility (TMT), inhibitory control (Stroop Colour Task), working memory (Digit Span) Long-term memory: simplified Taylor Complex Figure Test	Executive function was significantly improved in exercise group	Executive function: d = 1.31 Inhibitory control: d = 0.87 Working memory: d = 0.40 Cognitive flexibility: d = 0.96 Long-term memory: d = 0.22
	Ghodrati et al. [91]	Combined training: endurance + resistance + balance exercises	12 weeks	65 min	3x/week	Endurance: 55–75% HRmax resistance: 65–85% 1RM	Cognitive function (MoCA), information processing speed + overall cognitive functioning (DSST), short-term memory (forward digit span test)	Intervention group scored significantly higher on MoCA than control group, and increased score with 3.1 compared to baseline. No improvement in DSST or forward digit span test	MoCA: d = 0.18 DSST: d = 0.05 Forward digit span: d = 0.16
	Martinez-Velilla et al. [92]	Combination of resistance, balance, and walking exercises Squats rising from a chair, leg press, and bilateral knee extension, seated bench press semitandem foot standing, line walking, stepping practice, walking with small obstacles, proprioceptive exercises on unstable surfaces, altering the base of support, and weight transfer from one leg to the other, knee extension and flexion, hip abduction, walking along corridor	8 days	20 min	2x/day 5–7 days/week	?	MMSE	After the intervention, the exercise group scored on average 1.6 points higher than the control group (23.7 vs. 22.1), significant	MMSE: d = 0.69
	Callisaya et al. [93]	Combination of endurance and resistance exercise	6 months	1 h	3x/week	Started at low to moderate, progressed to moderate to vigorous	Cognitive function: Victoria Stroop test, TMT (shifting score B-A), the DSC Test, digit span subtest of the Wechsler Adult Intelligence Scale (third edition), Controlled Oral Word Association Test, three part HVLT-D (revised), Rey Complex Figure copy and delay	Better global cognitive score, better performance on the DSC Test, Rey Complex Copy test, Stroop C-D, Trail Making Test A and B, Hopkins intermediate and recognition, and Controlled Oral Word Association Test	Cognitive global score: d = 2.43 RCF copy: d = 1.67 RCF delay: d = 0.5 Stroop C-D: d = − 2.84 Trails B-A: d = − 0.48 DSC: d = 1.77 Digit span: d = − 3.43 Hopkins I: d =− 0.53 Hopkins D: d = 0.6 Hopkins R: d = 2 COWAT word: d = 1.57 COWAT category: d = 0.84
	Ghahfarrokhi et al. [94]	Endurance exercises, upper and lower body strength, balance exercises and maintaining posture, and hip control exercises and mid-body stability	6 weeks	30–35 min (HIFT) 40–45 min (LIFT)	3x/week (HIFT) 5x/week (LIFT)	HIFT: 100–120% lactate threshold LIFT: 70–75% lactate threshold	Cognitive screening (MMSE), processing speed (SDMT), learning (CVLT-II), memory (BVMT-R), attention (Stroop tests)	MMSE, Stroop, SDMT, CVLT-II and BVMT-R scores improved significantly in the HIFT group. MMSE and Stroop scores improved significantly in the LIFT group. Only change in Stroop scores in the HIFT group was significantly different from the control group	MMSE: HIFT: d = 0.73 LIFT: d = 0.71 Control: d = − 0.09 Stroop: HIFT: d = 0.82 LIFT: d = 0.72 Control: d = − 0.24 SDMT: HIFT: d = 0.55 LIFT: d = 0.22 Control: d = 0.08 CVLT-II: HIFT: d = 0.61 LIFT: d = 0.42 Control: d = 0.06 BVMT-R: HIFT: d = 0.66 LIFT: d = 0.45 Control: d = − 0.15
Other exercise types	Chen et al. [95]	24-form simplified Tai Chi Chuan	36 weeks	60 min	3x/week	Moderate intensity	Global cognition (MoCA), MQ, DSST, TMT-B, BNT, ROCFT	Both interventions significantly increased MoCA scores, with a significantly larger effect of Tai Chi Chuan compared to fitness walking. 24 weeks: DSST score was more effectively improved by the Tai Chi Chuan group compared to the fitness walking group. No significant differences in MQ, TMT-B, BNT or ROCFT between Tai Chi Chuan and fitness walking group. MoCA, MQ, DSST, and TMT-B scores improved significantly more in Tai Chi Chuan group compared to control group. MoCA scores in fitness walking group improved significantly more than in control group 36 weeks: MQ scores improved significantly more in Tai Chi Chuan group compared to fitness walking group. MQ, DSST, and TMT-B scores improved significantly more in Tai Chi Chuan group compared to control group. MoCA scores in fitness walking group improved significantly more than in control group	MoCA: Tai Chi Chuan vs. control: d = 0.63 Fitness walking vs. control: d = 0.33 Wechsler Memory Quotient (MQ): Tai Chi Chuan vs. control: d = 0.47 Fitness walking vs. control: d = 0.15 DSST: Tai Chi Chuan vs. control: d = 0.31 Fitness walking vs. control: d = 0.14 TMT B: Tai Chi Chuan vs. control: d = − 0.37 Fitness walking vs. control: d = − 0.06 BNT: Tai Chi Chuan vs. control: d = 0.21 Fitness walking vs. control: d = 0.16 ROCFT: Tai Chi Chuan vs. control: d = 0.07 Fitness walking vs. control: d = − 0.12 ROCFT delayed recall: Tai Chi Chuan vs. control: d = 0.01 Fitness walking vs. control: d = 0.00
	Cai et al. [96]	Kinect-based Kaimai-style Qigong	12 weeks	30 min	3x/week	Low intensity	Cognitive function (MMSE)	Qigong group showed significant improvements in cognitive function compared to the control group	MMSE: d = 1.75

*BNT* Boston Naming Test, *BVMT-R* Brief Visuospatial Memory Test-Revised, *CBS* Cambridge Brain Sciences, *CVLT-II* California Verbal Learning Test II, *d* cohen’s d, *DSC* digit symbol coding, *DSST* Digit Symbol Substitution Test, *HIFT* High-Intensity low-volume Functional Training, *HRmax* Heart Rate maximum, *HRR* heart rate reserve, *HVLT-D* Hopkins Verbal Learning Test revised, *I* rest interval between sets and strength-type exercises, *LIFT* Low-Intensity High-volume Functional Training, *MMSE* Mini-Mental State Examination, *MoCA* Montreal Cognitive Assessment, *MQ* Weschler Memory Quotient, *MRI* Magnetic Resonance Imaging, *MRs* maximal repetitions, *MTTS* Mental Test and Training System, *ROCFT* Rey-Osterrieth Complex Figure Test, *SDMT* Symbol Digit Modalities test, *TMT* Trail Making Test, *TUG* timed up-and-go, *V1* intervention midpoint, *V*2 postintervention, *v6MWT* velocity in 6 min walking test, ηp^2^ = partial eta-squared, 1RM = 1 Rep Maximum, 3MSE = Modified Mini Mental State Exam

**Table 4:** Intervention characteristics and outcomes of included animal studies

Author	Type of exercise training	Duration of exercise intervention	Duration of each training session	Frequency of exercise training	Intensity of exercise training	Outcomes (cognitive tests)	Main cognitive results	Effect size
Parsa et al. [98]	Swimming	12 weeks	40 min	5 days/week	?	Cognitive memory (novel object recognition test and elevated plus maze) Aversive memory and learning (passive avoidance learning test)	Swimming training alone insignificantly improved exploratory behaviour, locomotor activity, and passive avoidance memory, and non-spatial cognitive memory compared to sedentary diabetic rats	/
Shekarchian et al. [99]	Swimming	4 weeks	Week 1–2: 3 × 10 min Week 3–4: 6 × 10 min	5 days/week	?	Working memory (Y maze) Recognition memory (novel object recognition) Spatial memory (MWM)	Exercise improved all memory aspects in exercising T2DM mice compared to non-exercising T2DM mice	Elevated plus maze test: Percentage of spontaneous alternation: η^2^ = 0.02 Total arm entries: η^2^ = 0.01 Novel object recognition test: Familiar recognition ratio: η^2^ = 0.04 Novel recognition ratio: η^2^ = 0.08 MWM: Escape latency: η^2^ = 0.10 Platform quadrant time: η^2^ = 0.01 Platform crossings: η^2^ = 0.01
Jesmin et al. [100]	Treadmill running	4 months	30 min/day	5 days/week	Light intensity: OLETF 7.0 m/min, LETO 10.0 m/min; Moderate intensity: OLETF 12.5 m/min, LETO 20 m/min	Spatial learning memory and retention (MWM)	Shortened swim length and escape latency in both light and moderate exercise groups $\to$ improved memory	MWM: Escape latency: η^2^ = 0.11 Swim length: η^2^ = 0.08 Memory retention: η^2^ = 0.16
Lang et al. [101]	Treadmill running	8 weeks	37 min/day	5 days/week	Moderate intensity	Spatial learning and memory (MWM)	Compared with T group, the escape latency of E group was significantly reduced on day 4–5, treadmill exercise significantly increased the number of platform crossings in E group	MWM: Escape latency: d = − 1.39 Platform quadrant time: d = 1.01 Platform crossings: d = 1.32
Shima et al. [102]	Treadmill running	4 weeks	30 min/day	5 days/week	Moderate intensity (OLETF rats, 12.5 m/min; LETO rats, 20 m/min)	Memory (MWM)	Escape latency and swim path length of exercising OLETF rats were shortened, without alteration of the speed of swimming, at trials on days 2, 3 and 4 The time spent by OLETF rats in the platform area significantly improved after 4 weeks of exercise	MWM: Escape latency: η^2^ = 0.11 Swim length: η^2^ = 0.15 Platform quadrant time: η^2^ = 0.16
Shima et al. [103]	Running on a forced exercise wheel bed	4 weeks	30 min/day	5 days/week	Light intensity	Memory (MWM)	No effect of exercise on the escape latency, swim distance or speed in ob/ob mice. The times of crossing the target platform during the probe test in C57BL/6 mice were significantly greater than that in sedentary ob/ob mice, but not than that in exercised ob/ob mice	MWM: Swim length: η^2^ = 0.02 Swim duration: η^2^ = 0.05

*E group* diabetes + exercise group, *LETO* Long Evans Tokushima Otsuka, *MWM* Morris Water Maze, *ob/ob* obese-hyperglycemic, *OLETF* Otsuka Long-Evans Tokushima Fatty, *T group* diabetes group, *T2DM* type 2 diabetes Intervention characteristics and outcomes of included human studies Cognitive performance: Walking vs. control: d = 0.73 E-health vs. control: d = 1.15 Quality value: Walking vs. control: d = 0.34 E-health vs. control: d = 0.41 Continuity value: Walking vs. control: d = 0.63 E-health vs. control: d = 1.05 Verbal memory: Walking vs. control: d = 0.73 E-health vs. control: d = 1.70 Non-verbal memory: Walking vs. control: d = 6.57 E-health vs. control: d = 6.32 First 6 weeks: 40–50% HRR Last 6 weeks: 50–60% HRR MoCA: d = 0.46 MMSE: d = 0.05 Stroop: d = 0.12 TMT B: d = 0.03 MMSE: d = 0.58 MoCA: d = 0.55 moderate intensity: 40–59% HRR Executive function (dimensional change card sort test) Episodic memory (picture sequence memory test) Working memory (List sorting working memory test) Processing speed (Pattern Comparison Processing Speed Test) Executive function: η^2^ = 0.027 Episodic memory: η^2^ = 0.013 Working memory: η^2^ = 0.008 Processing speed: η^2^ = 0.045 Whole-body, machine based power training (3 sets of 8 reps of lateral pulldown, chest press, upper back, leg press, knee extension, and knee flexion, hip extension and hip abduction) High intensity (15–18 on Borg scale, 80% 1RM) MMSE: d = − 0.52 TMT A: d = 0.10 TMT B: d = 0.16 TMT B – TMT A: d = 0.31 Word list memory: d = − 0.04 Word list recall: d = 0.32 Word list recognition: d = 0.00 Body weight resistance training + exercises with elastic bands: tube fly, front raise, hammer curl, leg extension, calf raise, and squat Performed 20 times each Endurance exercise: elliptical, bike, treadmill, or upper-body cycle ergometer Resistance exercise Endurance: 60% HRmax resistance: 11–12 Borg scale The group with both T2DM and hypertension showed a significantly increased number of reactions, but no significantly improved reaction time. No differences were observed between the endurance and resistance exercise group 6 resistance exercises (reverse grip lat pulldown, leg press, bench press machine, calf raises machine, dumbbell shoulder press, and abdominal crunches) + 15 min of treadmill walking, with increasing weight and repetition number over 8 weeks week 1–2: 20 min at 100% v6MWT (aerobic) 2 × 13–15 MRs, i = 60 s (strength) week 3–4 20 min at 105% v6MWT (aerobic) 3 × 13–15 MRs, i = 60 s (strength) week 5–6 25 min at 105% v6MWT (aerobic) 3 × 10–12 MRs, i = 60 s (strength) week 7–8 25 min at 105–110% v6MWT (aerobic) 3 × 8–10 MRs, i = 60 s (strength) Memory: d = − 0.07 Verbal fluency: d = − 0.01 Processing speed: d = − 0.10 Attention/concentration: d = 0.64 Cognitive flexibility: d = 0.67 Inhibitory control: d = 0.89 Working memory: d = 0.88 3MSE: d = 3.24 DSC: d = 1.50 HVLT-D: d = 3.24 Executive function: d = 0.69 6 resistance exercises (reverse grip lat pulldown, leg press, bench press machine, calf raises machine, dumbbell shoulder press, and abdominal crunches) + treadmill walking, with increasing weight and repetition number over 8 weeks week 1–2: 20 min at 100% v6MWT (aerobic) 2 × 13–15 MRs, i = 60 s (strength) week 3–4 20 min at 105% v6MWT (aerobic) 3 × 13–15 MRs, i = 60 s (strength) week 5–6 25 min at 105% v6MWT (aerobic) 3 × 10–12 MRs, i = 60 s (strength) week 7–8 25 min at 105–110% v6MWT (aerobic) 3 × 8–10 MRs, i = 60 s (strength) Executive function: cognitive flexibility (TMT), inhibitory control (Stroop Colour Task), working memory (Digit Span) Long-term memory: simplified Taylor Complex Figure Test Executive function: d = 1.31 Inhibitory control: d = 0.87 Working memory: d = 0.40 Cognitive flexibility: d = 0.96 Long-term memory: d = 0.22 Endurance: 55–75% HRmax resistance: 65–85% 1RM MoCA: d = 0.18 DSST: d = 0.05 Forward digit span: d = 0.16 Combination of resistance, balance, and walking exercises Squats rising from a chair, leg press, and bilateral knee extension, seated bench press semitandem foot standing, line walking, stepping practice, walking with small obstacles, proprioceptive exercises on unstable surfaces, altering the base of support, and weight transfer from one leg to the other, knee extension and flexion, hip abduction, walking along corridor 2x/day 5–7 days/week Started at low to moderate, progressed to moderate to vigorous Cognitive global score: d = 2.43 RCF copy: d = 1.67 RCF delay: d = 0.5 Stroop C-D: d = − 2.84 Trails B-A: d = − 0.48 DSC: d = 1.77 Digit span: d = − 3.43 Hopkins I: d =− 0.53 Hopkins D: d = 0.6 Hopkins R: d = 2 COWAT word: d = 1.57 COWAT category: d = 0.84 30–35 min (HIFT) 40–45 min (LIFT) 3x/week (HIFT) 5x/week (LIFT) HIFT: 100–120% lactate threshold LIFT: 70–75% lactate threshold MMSE: HIFT: d = 0.73 LIFT: d = 0.71 Control: d = − 0.09 Stroop: HIFT: d = 0.82 LIFT: d = 0.72 Control: d = − 0.24 SDMT: HIFT: d = 0.55 LIFT: d = 0.22 Control: d = 0.08 CVLT-II: HIFT: d = 0.61 LIFT: d = 0.42 Control: d = 0.06 BVMT-R: HIFT: d = 0.66 LIFT: d = 0.45 Control: d = − 0.15 Both interventions significantly increased MoCA scores, with a significantly larger effect of Tai Chi Chuan compared to fitness walking. 24 weeks: DSST score was more effectively improved by the Tai Chi Chuan group compared to the fitness walking group. No significant differences in MQ, TMT-B, BNT or ROCFT between Tai Chi Chuan and fitness walking group. MoCA, MQ, DSST, and TMT-B scores improved significantly more in Tai Chi Chuan group compared to control group. MoCA scores in fitness walking group improved significantly more than in control group 36 weeks: MQ scores improved significantly more in Tai Chi Chuan group compared to fitness walking group. MQ, DSST, and TMT-B scores improved significantly more in Tai Chi Chuan group compared to control group. MoCA scores in fitness walking group improved significantly more than in control group MoCA: Tai Chi Chuan vs. control: d = 0.63 Fitness walking vs. control: d = 0.33 Wechsler Memory Quotient (MQ): Tai Chi Chuan vs. control: d = 0.47 Fitness walking vs. control: d = 0.15 DSST: Tai Chi Chuan vs. control: d = 0.31 Fitness walking vs. control: d = 0.14 TMT B: Tai Chi Chuan vs. control: d = − 0.37 Fitness walking vs. control: d = − 0.06 BNT: Tai Chi Chuan vs. control: d = 0.21 Fitness walking vs. control: d = 0.16 ROCFT: Tai Chi Chuan vs. control: d = 0.07 Fitness walking vs. control: d = − 0.12 ROCFT delayed recall: Tai Chi Chuan vs. control: d = 0.01 Fitness walking vs. control: d = 0.00 *BNT* Boston Naming Test, *BVMT-R* Brief Visuospatial Memory Test-Revised, *CBS* Cambridge Brain Sciences, *CVLT-II* California Verbal Learning Test II, *d* cohen’s d, *DSC* digit symbol coding, *DSST* Digit Symbol Substitution Test, *HIFT* High-Intensity low-volume Functional Training, *HRmax* Heart Rate maximum, *HRR* heart rate reserve, *HVLT-D* Hopkins Verbal Learning Test revised, *I* rest interval between sets and strength-type exercises, *LIFT* Low-Intensity High-volume Functional Training, *MMSE* Mini-Mental State Examination, *MoCA* Montreal Cognitive Assessment, *MQ* Weschler Memory Quotient, *MRI* Magnetic Resonance Imaging, *MRs* maximal repetitions, *MTTS* Mental Test and Training System, *ROCFT* Rey-Osterrieth Complex Figure Test, *SDMT* Symbol Digit Modalities test, *TMT* Trail Making Test, *TUG* timed up-and-go, *V1* intervention midpoint, *V*2 postintervention, *v6MWT* velocity in 6 min walking test, ηp^2^ = partial eta-squared, 1RM = 1 Rep Maximum, 3MSE = Modified Mini Mental State Exam Intervention characteristics and outcomes of included animal studies Cognitive memory (novel object recognition test and elevated plus maze) Aversive memory and learning (passive avoidance learning test) Week 1–2: 3 × 10 min Week 3–4: 6 × 10 min Working memory (Y maze) Recognition memory (novel object recognition) Spatial memory (MWM) Elevated plus maze test: Percentage of spontaneous alternation: η^2^ = 0.02 Total arm entries: η^2^ = 0.01 Novel object recognition test: Familiar recognition ratio: η^2^ = 0.04 Novel recognition ratio: η^2^ = 0.08 MWM: Escape latency: η^2^ = 0.10 Platform quadrant time: η^2^ = 0.01 Platform crossings: η^2^ = 0.01 MWM: Escape latency: η^2^ = 0.11 Swim length: η^2^ = 0.08 Memory retention: η^2^ = 0.16 MWM: Escape latency: d = − 1.39 Platform quadrant time: d = 1.01 Platform crossings: d = 1.32 Escape latency and swim path length of exercising OLETF rats were shortened, without alteration of the speed of swimming, at trials on days 2, 3 and 4 The time spent by OLETF rats in the platform area significantly improved after 4 weeks of exercise MWM: Escape latency: η^2^ = 0.11 Swim length: η^2^ = 0.15 Platform quadrant time: η^2^ = 0.16 MWM: Swim length: η^2^ = 0.02 Swim duration: η^2^ = 0.05 *E group* diabetes + exercise group, *LETO* Long Evans Tokushima Otsuka, *MWM* Morris Water Maze, *ob/ob* obese-hyperglycemic, *OLETF* Otsuka Long-Evans Tokushima Fatty, *T group* diabetes group, *T2DM* type 2 diabetes

## Abbreviations

Aβ: Amyloid β
AD: Alzheimer’s disease
AMPK: AMP-activated Protein Kinase
APOE: Apolipoprotein E
ATP: Adenosine Triphosphate
BACE1: β-Site AβPP-cleaving enzyme 1
BBB: Blood-brain barrier
BDNF: Brain-Derived Neurotrophic Factor
BNT: Boston Naming Test
BVMT-R: Brief Visuospatial Memory Test-Revised
CBS: Cambridge Brain Sciences
CIND: Cognitive impairment without dementia
CVLT-II: California Verbal Learning Test Second Edition
D: Cohen’d
DCX: Doublecortin
DLPF: Dorsolateral prefrontal cortex
DSC: Digit Symbol Coding
DSST: Digit Symbol Substitution Test
E group: Diabetes + exercise group
FNDC5: Fibronectin type III Domain-Containing protein 5
FOXO1: Forkhead box protein O1
FPA: Frontal pursuit area
GLP-1R: Glucagon-Like Peptide 1 Receptor
GLUT: Glucose transporter
GSK3β: Glycogen Synthase Kinase-3 beta
HIFT: High-intensity low-volume functional training
HRmax: Heart Rate maximum
HRR: Heart Rate Reserve
HVLT-D: Hopkins Verbal Learning Test revised
I: Rest interval between sets and strength-type exercises
ICE: Integrated concurrent exercise
IGF-1: Insulin-like-growth-factor 1
IGF-1R: Insulin-like-growth-factor 1 receptor
IR: Insulin receptor
IRS-1: Insulin Receptor Substrate 1
IL-1β: Interleukin 1beta
IL-6: Interleukin 6
LETO: Long-Evans Tokushima
LIFT: Low-intensity high-volume functional training
MCE: Multicomponent exercise
MCI: Mild cognitive impairment
MCT2: Monocarboxylate Transporter 2
MMSE: Mini mental state examination
MoCA: Montreal cognitive assessment
MQ: Weschler Memory Quotient
MRI: Magnetic Resonance Imaging
MRs: Maximal repetitions
MTTS: Mental test and training system
mTOR: Mammalian Target Of Rapamycin
MWM: Morris water maze
Nf-κB: Nuclear Factor kappa-light-chain-enhancer of activated B-cells
NMDAR: N-methyl-D-aspartate receptor
OFC: Orbitofrontal cortex
OLETF: Otsuka-Long-Evans-Tokushima fatty
PA: Physical activity
PGC-1α: Peroxisome proliferator-activated receptor Gamma Coactivator 1-alpha
PI3K: Phosphoinositide 3-Kinase
PKA: Protein Kinase A
PKC: Protein Kinase C
p-tau: Phosphorylated tau
ROCFT: Rey-Osterrieth Complex Figure Test
ROS: Reactive Oxygen Species
Ser-9: Serine-9
SD: Sprague-Dawley
SDMT: Symbol Digit Modalities Test
Sirt1: Sirtuin 1
SZDG: Subgranular zone of the dentate gyrus
SYN: Synaptophysin
T group: Diabetes group
TMT: Trail Making Test
TNF-α: Tumor Necrosis Factor alpha
TUG: Timed up-and-go
T2DM: Type 2 diabetes
VEGF: Vascular-endothelial growth factor
V1: Intervention midpoint
V2: Postintervention
v6MWT: Velocity in 6 min walking test
ZDF: Zucker diabetic fatty
ηp^2^: Partial eta-squared
1RM: 1 Rep maximum
3MSE: Modified Mini Mental State Exam
