# Supplementary material for: The Impact of Exercise Training on the Brain and Cognition in Type 2 Diabetes, and its Physiological Mediators: A Systematic Review
Source: Sports Med Open. 2025 Apr 24;11:42. doi: 10.1186/s40798-025-00836-7 (PMC12022206; doi:10.1186/s40798-025-00836-7)
Supplement: Supplementary file 1 — Additional file 1. [file 40798_2025_836_MOESM1_ESM.docx]

**Supplementary table 1 Population characteristics of included human studies**

|  | **Author** | **Year** | **Age** | **Population** | **N intervention** | **Control group** | **N controls** |
| --- | --- | --- | --- | --- | --- | --- | --- |
| **Endurance exercise training** | Leischik et al.[1] | 2021 | 59.1 (control); 60.4 (walking); 56.4 (pedometer) | T2DM patients walking or T2DM patients counting 10 000 steps/day with pedometer | 36  (18 per group) | T2DM patients receiving standard care | 16 |
|  | Ploydang et al.[2] | 2023 | 60-75 | T2DM patients receiving endurance training | 17 | T2DM patients without exercise training | 17 |
|  | Wang et al.[3] | 2023 | 60-75 | T2DM patients receiving endurance training | 40 | T2DM patients without exercise training | 42 |
|  | Liu et al.[4] | 2024 | 56.84 | T2DM patients walking | 33 | T2DM patients stretching | 17 |
| **Resistance exercise training** | Zhao et al.[5] | 2022 | 67.9 ± 5.5 | T2DM patients receiving power training | 49 | T2DM patients with sham low-intensity training (same machines: 3 sets of 8 reps with empty bar) | 54 |
|  | Yamamoto et al.[6] | 2021 | 72.9 ± 2.4 | T2DM patients performing resistance exercise or resistance exercise + leucine supplementation | 18 (R) 17 (RL) | T2DM patients without exercise training | 17 |
| **Endurance vs. resistance exercise training** | Teixeira et al.[7] | 2019 | 55 ± 12 | Patients with T2DM and/or systemic arterial hypertension receiving resistance training | 6 | Patients with T2DM and/or systemic arterial hypertension receiving endurance training | 7 |
| **Combined endurance and resistance exercise training** | Silveira-Rodrigues et al.[8] | 2021 | 63.9 ± 7.7 (training)  63.3 ± 7.8 (control) | T2DM patients receiving combined endurance and resistance exercise training | 16 | T2DM patients without exercise training | 15 |
|  | Espeland et al.[9] | 2017 | 70-89 | Sedentary non-demented T2DM patients and non-diabetic adults receiving physical activity intervention | 735 | Sedentary non-demented T2DM patients and non-diabetic adults receiving health education | 741 |
|  | Silveira-Rodrigues et al.[10] | 2023 | 63±8 | T2DM patients receiving combined exercise training | 17 | T2DM patients without exercise training | 18 |
|  | Ghodrati et al.[11] | 2023 | 55-70 | Women with T2DM receiving combined exercise training | 13 | Women with T2DM without exercise training | 9 |
|  | Martinez-Velilla et al.[12] | 2021 | 86 ± 5 (control) 87 ± 4 (intervention) | Acutely hospitalised elderly T2DM patients receiving combined exercise training | 54 | acutely hospitalised elderly T2DM patients receiving usual care | 49 |
|  | Callisaya et al.[13] | 2017 | 66.2 | T2DM patients receiving a multi-modal exercise program | 24 | T2DM patients who received upper and lower limb stretching of light intensity and a gentle movement program, which were performed in the same volume, frequency and setting as the intervention group | 23 |
|  | Ghahfarrokhi et al.[14] | 2024 | 67.5 ± 5.8 | Elderly, cognitively impaired T2DM patients performing HIFT or LIFT | 32  (16 per group) | Elderly, cognitively impaired T2DM patients without intervention | 16 |
| **Other exercise types** | Chen et al.[15] | 2023 | 67.55 [5.02] | T2DM patients performing Tai Chi Chuan | 107 | T2DM patients performing fitness walking + control group receiving only self-management education | 110 (fitness walking) 111 (control) |
|  | Cai et al.[16] | 2019 | older adults | T2DM patients performing Qigong exercise | 27 | T2DM patients without intervention | 28 |

*HIFT = high-intensity low-volume functional training, LIFT = low-intensity high-volume functional training, R = resistance exercise, RL = resistance exercise + leucine supplementation, T2DM = type 2 diabetes*

**Supplementary table 2 Population characteristics of included animal studies**

| **Author** | **Year** | **Age** | **Population** | **N intervention** | **Control group** | **N controls** |
| --- | --- | --- | --- | --- | --- | --- |
| Parsa et al.[17] | 2021 | 6-8 weeks | Male diabetic albino Wistar rats with swimming training, P. Psyllium, or both | 30 (10 per group) | Healthy sedentary male albino Wistar rats, sedentary diabetic male albino Wistar rats | 20 (10 per group) |
| Shekarchian et al.[18] | 2023 | 10-11 weeks | Exercising adult male C57BL/6 J T2DM mice | 10 | Non-exercising adult male C57BL/6 J T2DM and control mice + exercising adult male C57BL/6 J control mice | 30  (10 per group) |
| Jesmin et al.[19] | 2022 | 8 weeks | OLETF rats | 26 | LETO rats (sedentary, light intensity exercise, and moderate intensity exercise) + sedentary OLETF rats | 38  (13, 7, 5 and 13 respectively) |
| Lang et al.[20] | 2020 | 10 weeks | Exercising C57BL/ 6 T2DM mice | 9 | Control C57BL/6 mice, non-exercising T2DM mice, and exercising T2DM mice with nicotinamide treatment | 27  (9 per group) |
| Shima et al.[21] | 2017 | 26 weeks | Exercising OLETF rats | 6 to 8 | Exercising and non-exercising LETO rats + non-exercising OLETF rats | 6 to 8 per group |
| Shima et al.[22] | 2023 | 8 weeks | Exercising ob/ob mice | 5 | Sedentary ob/ob mice and sedentary C57BL/6 mice | 10 (5 per group) |

*LETO = Long-Evans Tokushima, ob/ob = obese-hyperglycemic, OLETF = Otsuka-Long-Evans-Tokushima fatty, T2DM = type 2 diabetes*

1. Leischik, R., et al., *Exercise Improves Cognitive Function-A Randomized Trial on the Effects of Physical Activity on Cognition in Type 2 Diabetes Patients.* J Pers Med, 2021. **11**(6).

2. Ploydang, T., et al., *Nordic Walking in Water on Cerebrovascular Reactivity and Cognitive Function in Elderly Patients with Type 2 Diabetes.* Med Sci Sports Exerc, 2023.

3. Wang, Y., et al., *Aerobic Training Increases Hippocampal Volume and Protects Cognitive Function for Type 2 Diabetes Patients with Normal Cognition.* Exp Clin Endocrinol Diabetes, 2023.

4. Liu, T., et al., *The effects of aerobic exercise on cognitive function in middle-aged and older individuals with type 2 diabetes: A pilot randomized controlled trial.* Geriatr Nurs, 2024. **60**: p. 677-685.

5. Zhao, R.R., et al., *Effect of High-Intensity Power Training on Cognitive Function in Older Adults With Type 2 Diabetes: Secondary Outcomes of the GREAT2DO Study.* J Gerontol A Biol Sci Med Sci, 2022. **77**(10): p. 1975-1985.

6. Yamamoto, Y., et al., *Effects of resistance training using elastic bands on muscle strength with or without a leucine supplement for 48 weeks in elderly patients with type 2 diabetes.* Endocr J, 2021. **68**(3): p. 291-298.

7. Teixeira, R.B., et al., *Evaluating the effects of exercise on cognitive function in hypertensive and diabetic patients using the mental test and training system.* World J Biol Psychiatry, 2019. **20**(3): p. 209-218.

8. Silveira-Rodrigues, J.G., et al., *Combined exercise training improves specific domains of cognitive functions and metabolic markers in middle-aged and older adults with type 2 diabetes mellitus.* Diabetes Res Clin Pract, 2021. **173**: p. 108700.

9. Espeland, M.A., et al., *Effects of Physical Activity Intervention on Physical and Cognitive Function in Sedentary Adults With and Without Diabetes.* J Gerontol A Biol Sci Med Sci, 2017. **72**(6): p. 861-866.

10. Silveira-Rodrigues, J.G., et al., *Combined Training Improves Executive Functions Without Changing Brain-Derived Neurotrophic Factor Levels of Middle-Aged and Older Adults with Type 2 Diabetes.* Exp Clin Endocrinol Diabetes, 2023. **131**(6): p. 345-353.

11. Ghodrati, N., et al., *Effect of Combined Exercise Training on Physical and Cognitive Function in Women With Type 2 Diabetes.* Can J Diabetes, 2023. **47**(2): p. 162-170.

12. Martinez-Velilla, N., et al., *Effects of a Tailored Exercise Intervention in Acutely Hospitalized Oldest Old Diabetic Adults: An Ancillary Analysis.* J Clin Endocrinol Metab, 2021. **106**(2): p. e899-e906.

13. Callisaya, M.L., et al., *Feasibility of a multi-modal exercise program on cognition in older adults with Type 2 diabetes - a pilot randomised controlled trial.* BMC Geriatr, 2017. **17**(1): p. 237.

14. Ghahfarrokhi, M.M., et al., *Feasibility and preliminary efficacy of different intensities of functional training in elderly type 2 diabetes patients with cognitive impairment: a pilot randomised controlled trial.* BMC Geriatr, 2024. **24**(1): p. 71.

15. Chen, Y., et al., *Effects of Tai Chi Chuan on Cognitive Function in Adults 60 Years or Older With Type 2 Diabetes and Mild Cognitive Impairment in China: A Randomized Clinical Trial.* JAMA Netw Open, 2023. **6**(4): p. e237004.

16. Cai, H., et al., *Effect of Low-Intensity, Kinect-Based Kaimai-Style Qigong Exercise in Older Adults With Type 2 Diabetes.* J Gerontol Nurs, 2019. **45**(2): p. 42-52.

17. Parsa, H., et al., *Swimming training and Plantago psyllium ameliorate cognitive impairment and glucose tolerance in streptozotocin-nicotinamide-induced type 2 diabetic rats.* J Physiol Sci, 2021. **71**(1): p. 37.

18. Shekarchian, M., M. Peeri, and M.A. Azarbayjani, *Physical activity in a swimming pool attenuates memory impairment by reducing glutamate and inflammatory cytokines and increasing BDNF in the brain of mice with type 2 diabetes.* Brain Res Bull, 2023. **201**: p. 110725.

19. Jesmin, S., et al., *Long-term light and moderate exercise intervention similarly prevent both hippocampal and glycemic dysfunction in presymptomatic type 2 diabetic rats.* Am J Physiol Endocrinol Metab, 2022. **322**(3): p. E219-E230.

20. Lang, X., et al., *Treadmill exercise mitigates neuroinflammation and increases BDNF via activation of SIRT1 signaling in a mouse model of T2DM.* Brain Res Bull, 2020. **165**: p. 30-39.

21. Shima, T., et al., *Moderate exercise ameliorates dysregulated hippocampal glycometabolism and memory function in a rat model of type 2 diabetes.* Diabetologia, 2017. **60**(3): p. 597-606.

22. Shima, T., et al., *Light-intensity exercise improves memory dysfunction with the restoration of hippocampal MCT2 and miRNAs in type 2 diabetic mice.* Metab Brain Dis, 2023. **38**(1): p. 245-254.
